# Derivation of the Landau–Pekar Equations in a Many-Body Mean-Field Limit

**DOI:** 10.1007/s00205-021-01616-9

**Published:** 2021-02-26

**Authors:** Nikolai Leopold, David Mitrouskas, Robert Seiringer

**Affiliations:** 1 Department of Mathematics and Computer Science, University of Basel, Spiegelgasse 1, 4051 Basel, Switzerland; 2 Fachbereich Mathematik, Universität Stuttgart, Pfaffenwaldring 57, 70569 Stuttgart, Germany; 3 Present Address: Institute of Science and Technology Austria (IST Austria), Am Campus 1, 3400 Klosterneuburg, Austria

## Abstract

We consider the Fröhlich Hamiltonian in a mean-field limit where many bosonic particles weakly couple to the quantized phonon field. For large particle numbers and a suitably small coupling, we show that the dynamics of the system is approximately described by the Landau–Pekar equations. These describe a Bose–Einstein condensate interacting with a classical polarization field, whose dynamics is effected by the condensate, i.e., the back-reaction of the phonons that are created by the particles during the time evolution is of leading order.

## Introduction

We consider the dynamics of *N* bosonic particles interacting with a quantized phonon field described by the Fröhlich model in a mean field regime. The underlying Hilbert space is

1.1 $$\begin{aligned} {\mathcal {H}}^{(N)} = L^2_s\left( {\mathbb {R}}^{3N} \right) \otimes {\mathcal {F}}_s , \end{aligned}$$

where the *N* particles are described by states in $L^2_s({\mathbb {R}}^{3N})$, the subspace of all complex-valued square integrable *N*-particle wave functions that are symmetric under the exchange of any pair of the coordinates $(x_1,...,x_N)$, and where the phonon field is represented by elements in the bosonic Fock space ${\mathcal {F}}_s = \bigoplus _{n \geqq 0} L^2_s({\mathbb {R}}^{3n}).$ The time evolution of the system is governed by the Schrödinger equation

1.2 $$\begin{aligned} i \partial _t \Psi _{N,t} = H_{N,\alpha }^\mathrm{F} \Psi _{N,t}, \end{aligned}$$

with the Fröhlich Hamiltonian

1.3 $$\begin{aligned} H_{N,\alpha }^\mathrm{F}&= \sum _{j=1}^N \left[ - \Delta _j + \sqrt{\frac{\alpha }{N}} \int d^3k \, \left| k \right| ^{-1} \left( e^{ikx_j} a_k + e^{-ikx_j } a^*_k \right) \right] + {\mathcal {N}} . \end{aligned}$$

Here, $\Delta _j$ is the Laplacian acting on the *j*th particle with coordinate $x_j$, $a_k$ and $a_k^*$ denote the usual bosonic annihilation and creation operators satisfying the canonical commutation relations

1.4 $$\begin{aligned}{}[a_k, a^*_l ]&= \delta (k-l), \quad [a_k, a_l ] = [a^*_k, a^*_l ] = 0, \end{aligned}$$

and ${\mathcal {N}}$ is the number operator defined by ${\mathcal {N}}= \int d^3 k\, a_k^* a_k$. The coupling parameter $\sqrt{\alpha /N}$ is introduced to scale the strength of the interaction between the particles and the phonon field. If the number of phonons is of order *N* and $\alpha >0$ is fixed, the factor $N^{-1/2}$ and the fact that the creation and annihilation operators scale like $\sqrt{N}$ (they are bounded by $\left( {\mathcal {N}} + 1 \right) ^{1/2}$, see (3.3)) ensure that the kinetic and potential energy are of the same order for large *N*.

We note that the expression (1.3) is somewhat formal, since the form factor $\vert k\vert ^{-1}$ in the interaction term is not square integrable. By a well-known argument going back to Lieb and Yamazaki [26] (cf. Lemma A.1), the right side of (1.3) defines a closed bounded from below quadratic form with domain given by the form domain of $H_{N,0}^\mathrm{F}$. The self-adjoint operator that corresponds to this form is called Fröhlich Hamiltonian and denoted by $H_{N,\alpha }^\mathrm{F}$. We refer to [16] for a detailed description of its domain ${\mathcal {D}}(H_{N,\alpha }^\mathrm{F})$ (see also Lemma 3.1).

If the number *N* of particles is large, we show for a particular class of initial states that the solution of the many-body Schrödinger equation (1.2) can be approximated by Pekar product states, i.e., states of the form

1.5 $$\begin{aligned} \Psi _{N,t} = \psi _t^{\otimes N}\otimes W(\sqrt{N} \varphi _t) \Omega , \end{aligned}$$

where $\Omega$ is the vacuum state in ${\mathcal {F}}_s$, *W* denotes the Weyl operator and $(\psi _t,\varphi _t) \in L^2({\mathbb {R}}^3) \times L^2({\mathbb {R}}^3)$ solve the time-dependent Landau–Pekar equations

1.6 $$\begin{aligned} {\left\{ \begin{array}{ll} i \partial _t \psi _t(x) &{} = \ \ \left[ - \Delta _x + \sqrt{ \alpha } \Phi (x,t) \right] \psi _t(x), \\ i \partial _t \varphi _t(k) &{} = \ \ \varphi _t(k) + \sqrt{ \alpha } \left| k \right| ^{-1} \int d^3x \, e^{-ikx} \left| \psi _t(x) \right| ^2 \end{array}\right. }, \end{aligned}$$

where

1.7 $$\begin{aligned} \Phi (x,t)&= \int d^3k \, \left| k \right| ^{-1} \left( e^{ikx} \varphi _t(k) + e^{-ikx} \overline{\varphi _t(k)} \right) . \end{aligned}$$

The Weyl operator is defined for any $f\in L^2({\mathbb {R}}^3)$ by

1.8 $$\begin{aligned} W(f) \!=\! \exp \left( \int d^3k \, \big ( f(k) a^*_k \!-\! \overline{f(k)} a_k \big ) \right) . \end{aligned}$$

In the Pekar product state (1.5), the phonons are in the coherent state $W (\sqrt{N} \varphi _t) \Omega$ with average number of excitations of order *N*, and the bosonic particles form a pure Bose–Einstein condensate with condensate wave function $\psi _t$. According to the Landau–Pekar equations, the one-particle condensate wave function $\psi _t$ evolves in the potential $\sqrt{\alpha }\Phi (x,t)$ created by the phonons, while the phonon field couples to the particles via the source term involving the density $\left| \psi _t(x) \right| ^2$.

Our main result can be summarized as follows: given an initial wave function $\Psi _{N,0}$ that is close to a Pekar product state $\psi _0^{\otimes N}\otimes W(\sqrt{N} \varphi _0)\Omega$ (close in an appropriate sense that will be specified in the next section), then the time evolved state $e^{-iH_{N,\alpha }^\mathrm{F} t}\Psi _{N,0}$ remains close to the time evolved Pekar state (1.5) when $N\gg 1$.

The Landau–Pekar equations were originally introduced in [21] to approximate the time evolution of a single polaron in the strong coupling limit. In our notation, the strong coupling regime corresponds to the Hamiltonian $H_{1,\alpha }^\mathrm{F}$ with $\alpha \gg 1$. Partial results concerning a rigorous derivation of the Landau–Pekar equations in the strong coupling limit were obtained in [8, 10, 15, 25] (for a detailed comparison between the different results we refer to [25, Chapter 2]). In these works, the Landau–Pekar equations are justified for short times, namely at most for times of order $\alpha ^{-\varepsilon }$ with $\varepsilon >0$ arbitrary small.^1^ A derivation for times of order one, the time scale in the strong coupling limit at which the back-reaction of the phonons that are created during the time evolution is of leading order, remains an open problem. The emergence of classical radiation in the strong coupling limit is expected to rely on the adiabatic decoupling between the relatively fast moving (with respect to $\alpha$) electron and the radiation field. For results on adiabatic theorems of the Landau–Pekar equations in one and three dimensions we refer to [9, 25].

In the many-particle mean-field limit considered in this work, the creation of coherent radiation happens for a different reason than in the strong coupling regime, namely because there are many particles in the same quantum state that simultaneously create the phonons. In this regard, the present work is related to [1, 7, 22–24], where many-body mean-field limits of the renormalized Nelson model, the Nelson model with ultraviolet cutoff and the (bosonic) Pauli–Fierz model are considered. In particular, we mention [1] where the Schrödinger–Klein–Gordon equations were derived by the Wigner measure approach as a limit of the renormalized Nelson model.

In [4, 5, 12], effective equations for the Nelson, Pauli–Fierz and Fröhlich model were derived in a partially classical limit. There, the number of particles is kept fixed while the number of excitations of the quantum field tends to infinity and the coupling constant approaches zero in a suitable sense. The effect of the excitations that are created during time evolution is negligible in this limit and the quantum field can thus be approximated by a classical field that evolves freely or remains constant in time.

To the best of our knowledge, the present work provides the first derivation of the Landau–Pekar equations in a limit in which the back-reaction of the phonons that are created during time evolution is of leading order. Moreover, our results include explicit error estimates.

In order to derive our results, we follow [24], which combines the methods from [29, 31]. The new technical challenge in comparison with [24] is to show that the high momentum phonons do not obstruct the expected mean-field behavior. This requires several nontrivial modifications. First, it is crucial to introduce a measure for the excitations around the condensate resp. around the coherent state that involves the canonical transformation due to Gross and Nelson (see (2.12)). In particular, we use the representation of the Fröhlich Hamiltonian in [16]. The most difficult part is to control the interaction between the ultraviolet modes of the phonon field and the fraction of particles not in the condensate. To this end, we restrict our consideration to a subclass of the initial states which have small fluctuations in the energy per particle observable and combine estimates similar to [23, Sect. VIII.1] with an operator bound that is motivated by [10, Lemma 10]. The idea of using this restriction in order to treat the singular interaction between quantum fields and particles in the mean field regime was already used in [23].

The article is organized as follows: in the next section, we state our main results. In Theorem 2.1, we consider initial states in the domain of the Fröhlich Hamiltonian, while Theorem 2.2 is about initial states in the domain of the noninteracting model (including, in particular, product states). In Section 3, we introduce useful notation and discuss the representation of the Fröhlich Hamiltonian via the Gross transformation. The key steps of the proof of our main result are summarized in Section 4 in terms of several lemmas. The proofs of these are given in Sections 5–7.

## Main Results

For notational convenience, we set the coupling constant $\alpha = 1$ from now on and denote $H_N^\mathrm{F} = H_{N,1}^\mathrm{F}$. All statements and proofs that follow are, however, equally true for any $\alpha >0$ independent of *N*.

In order to state our main results we define for $\Psi _N\in {\mathcal {H}}^{(N)}$ the one-particle reduced density matrix

2.1 $$\begin{aligned} \gamma _{\Psi _N}^{(1,0)} = \text {Tr}_{2,\ldots , N} \otimes \text {Tr}_{{\mathcal {F}}_s} | \Psi _{N} \rangle \langle \Psi _{N} | \end{aligned}$$

on the Hilbert space $L^2({\mathbb {R}}^3)$. Here, $\text {Tr}_{2,\ldots , N}$ denotes the partial trace over the coordinates $x_2,\ldots , x_N$ and $\text {Tr}_{{\mathcal {F}}_s}$ the trace over Fock space. The particles of a many-body state $\Psi _{N}$ are said to exhibit complete Bose–Einstein condensation if there exists $\psi \in L^2({\mathbb {R}}^3)$ with $\left\| \psi \right\| _{L^2({\mathbb {R}}^3)}=1$ such that

2.2 $$\begin{aligned} \text {Tr}_{L^2({\mathbb {R}}^3)} \left| \gamma _{\Psi _N}^{(1,0)} - | \psi \rangle \langle \psi | \right| \rightarrow 0 \end{aligned}$$

as $N \rightarrow \infty$. In this case $\psi$ is called the condensate wave function. Moreover, we define (for $\left( \psi , \varphi \right) \in L^2({\mathbb {R}}^3) \times L^2({\mathbb {R}}^3)$ and $\Psi _{N}\in {\mathcal {D}} \left( H_N^\mathrm{F} \right)$)

2.3 $$\begin{aligned} a(\Psi _{N},\psi )&= \text {Tr}_{L^2({\mathbb {R}}^3)} \left| \gamma ^{(1,0)}_{\Psi _{N }} - | \psi \rangle \langle \psi | \right| , \end{aligned}$$

2.4 $$\begin{aligned} b(\Psi _{N},\varphi )&= N^{-1} \big \langle W^* (\sqrt{N} \varphi ) \Psi _{N } , {\mathcal {N}} \, W^* (\sqrt{N} \varphi ) \Psi _{N} \big \rangle , \end{aligned}$$

2.5 $$\begin{aligned} c(\Psi _{N})&= \left\| N^{-1} \left( H_N^\mathrm{F} - \big \langle \Psi _{N} , H_N^\mathrm{F} \Psi _{N} \big \rangle \right) \Psi _{N} \right\| ^2 . \end{aligned}$$

For $m \in {\mathbb {N}}$, let $H^m({\mathbb {R}}^3)$ denote the Sobolev space of order *m* and $L_m^2({\mathbb {R}}^3)$ a weighted $L^2$-space with norm $\Vert \varphi \Vert _{L_m^2({\mathbb {R}}^3)} = \Vert ( 1 + \left| \,\cdot \, \right| ^2 )^{m/2} \varphi \Vert _{L^2({\mathbb {R}}^3)}$. We will use the following result which was proven in [8]:

### Proposition 2.1

(Lemma C.2 in [8]) The Landau–Pekar equations (1.6) are globally well-posed in $H^2({\mathbb {R}}^3) \times L_1^2({\mathbb {R}}^3)$. For all $t \in {\mathbb {R}}$ we have

2.6 $$\begin{aligned} \left\| \psi _t \right\| _{H^2({\mathbb {R}}^3)}&\leqq C \left( 1 + \left| t \right| \right) \quad \text {and} \quad \left\| \varphi _t \right\| _{L_1^2({\mathbb {R}}^3)} \leqq C \left( 1 + \left| t \right| \right) , \end{aligned}$$

where *C* is a constant depending only on the initial data.

We are now ready to state our main theorem.

### Theorem 2.1

Let $(\psi , \varphi ) \in H^2({\mathbb {R}}^3) \times L_1^2({\mathbb {R}}^3)$ s.t. $\left\| \psi \right\| _{L^2({\mathbb {R}}^3)} = 1$, and $\Psi _{N} \in {\mathcal {D}} ( H_N^\mathrm{F} )$ s.t. $\left\| \Psi _{N} \right\| = 1$ and $E_0 = \sup _{N \in {\mathbb {N}}} \big \vert N^{-1} \big \langle \Psi _{N } , H_N^\mathrm{F} \Psi _{N } \big \rangle \big \vert <\infty$. Let $(\psi _t, \varphi _t)$ be the unique solution of (1.6) with initial datum $(\psi , \varphi )$ and $\Psi _{N,t}= e^{-iH_N^\mathrm{F} t}\Psi _{N}$. Then, there exists a constant $C >0$ (depending only on $\left\| \varphi \right\| _{L_1^2({\mathbb {R}}^3)}$, $\left\| \psi \right\| _{H^2({\mathbb {R}}^3)}$ and $E_0$) such that

2.7 $$\begin{aligned} \text {Tr}_{L^2({\mathbb {R}}^3)} \left| \gamma ^{(1,0)}_{\Psi _{N,t}} - | \psi _t \rangle \langle \psi _t | \right|&\leqq \sqrt{ a(\Psi _{N},\psi ) + b(\Psi _{N},\varphi ) + c(\Psi _{N}) + N^{-1/2}} e^{C (1+\left| t \right| )^3 } , \end{aligned}$$

2.8 $$\begin{aligned} N^{-1} \big \langle W^* (\sqrt{N} \varphi _t) \Psi _{N,t} ,&{\mathcal {N}} \, W^* (\sqrt{N} \varphi _t) \Psi _{N,t} \big \rangle \nonumber \\ {}&\leqq \left( a(\Psi _{N},\psi ) + b (\Psi _{N },\varphi ) + c(\Psi _{N }) + N^{-1/2} \right) e^{C ( 1 + \left| t \right| )^3 } . \end{aligned}$$

The proof is given in Section 4.

### Remark 2.1

If one considers initial many-body states in which the particles are in a Bose–Einstein condensate, the phonons are in a coherent states and the energy has small fluctuations around its mean value, i.e.

2.9 $$\begin{aligned} \lim _{N \rightarrow \infty } \left( a(\Psi _{N},\psi ) + b(\Psi _{N},\varphi ) + c(\Psi _{N}) \right) = 0, \end{aligned}$$

it follows from Theorem 2.1 that

2.10 $$\begin{aligned} \lim _{N \rightarrow \infty } \text {Tr}_{L^2({\mathbb {R}}^3)} \left| \gamma ^{(1,0)}_{\Psi _{N,t}} - | \psi _t \rangle \langle \psi _t | \right|&=0 \quad \text {and} \nonumber \\ \lim _{N \rightarrow \infty } N^{-1} \big \langle W^* (\sqrt{N} \varphi _t) \Psi _{N,t} , {\mathcal {N}} \, W^* (\sqrt{N} \varphi _t) \Psi _{N,t} \big \rangle&=0 . \end{aligned}$$

Our result consequently shows the stability of the condensate and the coherent state during the time evolution.

### Remark 2.2

The condition $c(\Psi _{N}) \rightarrow 0$ as $N \rightarrow \infty$ restricts the initial data to many-body states $\Psi _{N}$ whose energy per particle has small fluctuations around its mean value. In our proof, this is important to obtain sufficient control on the singular ultraviolet behavior of the interaction term in $H_N^\mathrm{F}$. We give a detailed explanation of this point in Section 5. In the presence of an ultraviolet cutoff in the Fröhlich Hamiltonian, the estimates (2.7) and (2.8) hold without the appearance of $c(\Psi _{N})$ on the right hand side, but with a cutoff dependent constant *C*. In this simpler case, the statement could be proven in close analogy to [7, 24] where the Nelson model was considered with ultraviolet cutoff.

Next, we give examples of initial states that satisfy (2.9). The quantities $a(\Psi _N,\psi )$ and $b(\Psi _N,\varphi )$ are identically zero for Pekar product states $\Psi _{N}=\psi ^{\otimes N} \otimes W(\sqrt{N} \varphi ) \Omega$ with $(\psi , \varphi ) \in H^2({\mathbb {R}}^3) \times L_1^2({\mathbb {R}}^3)$. However, such Pekar states are in the domain ${\mathcal {D}}(H_N^0) = \big ( H_s^2({\mathbb {R}}^{3N}) \otimes {\mathcal {F}}_s \big ) \cap {\mathcal {D}} ({\mathcal {N}} )$ of the free Hamiltonian

2.11 $$\begin{aligned} H_N^0 = -\sum _{j=1}^N \Delta _{j} + {\mathcal {N}}, \end{aligned}$$

and thus, as shown in [16], can not be elements of ${\mathcal {D}} ( H_N^\mathrm{F} )$. As a consequence, $c(\Psi _{N})$ would be infinite in this case. To specify states that satisfy (2.9), we introduce the Gross transform

2.12 $$\begin{aligned} U_{K} = \exp \Bigg [ N^{-1/2} \sum _{j=1}^N \int d^3k \, \left( \overline{B_{K,x_j}(k)} a_k - B_{K,x_j}(k) a_k^* \right) \Bigg ], \end{aligned}$$

where

2.13 $$\begin{aligned} B_{K,x }(k)&= \frac{-1}{\left| k \right| (1 + k^2)} e^{-ikx } \mathbb {1}_{\left| k \right| \geqq K}(k) \end{aligned}$$

for $0< K< \infty$. The Gross transform, which goes back to Gross and Nelson [17, 28], relates the domains of $H_N^0$ and $H_N^\mathrm{F}$ to each other.^2^ In Lemma 3.1 we show that there is a ${{\widetilde{K}}}>0$ such that for all $K\geqq {{\widetilde{K}}}$ and all $N\geqq 1$, the domains satisfy

2.14 $$\begin{aligned} {\mathcal {D}} \left( H^\mathrm{F}_N \right)&= U_K^* {\mathcal {D}} \left( H_N^0 \right) . \end{aligned}$$

If we choose *K* as an *N*-dependent sufficiently rapidly growing sequence $(K_N)_{N\geqq 1}$, then the Gross transform $U_{K_N}$ has negligible effect on the condensate and the coherent state structure. This is summarized in the next proposition.

### Proposition 2.2

Assume $$K\geqq c $$ for some $$c > 0 $$ and consider the state $$\Psi _{N} = U_K^*\ \big ( \psi ^{\otimes N} \otimes W(\sqrt{N} \varphi ) \Omega \big )$$ with $$(\psi , \varphi ) \in H^2({\mathbb {R}}^3) \times L_1^2({\mathbb {R}}^3)$$ and $$\left\| \psi \right\| _{L^2({\mathbb {R}}^3)} = 1$$. Then there exists a $$C>0$$ such that $$\sup _{N\in {\mathbb {N}}} \big \vert N^{-1}\big \langle \Psi _{N} , H_N^\mathrm {F} \Psi _{N} \big \rangle \big \vert \leqq C$$ and

2.15 $$\begin{aligned} a(\Psi _{N},\psi )&\leqq \frac{C}{K^{3/2}},\quad b(\Psi _{N},\varphi ) \leqq \frac{C}{K^3}, \quad c(\Psi _{N}) \leqq C \Big ( K^{-1} + N^{-1} + \frac{K}{N^2} \Big ) \end{aligned}$$

with $a(\Psi _{N},\psi )$, $b(\Psi _{N},\varphi )$ and $c(\Psi _N)$ defined as in Theorem 2.1.

We prove this proposition in Section 7.2. As an immediate consequence of Proposition 2.2 (with $K=cN$) and Theorem 2.1 one finds

2.16 $$\begin{aligned} \text {Tr}_{L^2({\mathbb {R}}^3)} \left| \gamma ^{(1,0)}_{\Psi _{N,t}} - | \psi _t \rangle \langle \psi _t | \right| \leqq N^{-1/4} e^{C ( 1+ \left| t \right| )^3 } \end{aligned}$$

and

2.17 $$\begin{aligned} \frac{1}{N} \big \langle W^*(\sqrt{N} \varphi _t) \Psi _{N,t} , {{\mathcal {N}}} \, W^* (\sqrt{N} \varphi _t) \Psi _{N,t} \big \rangle \leqq N^{-1/2} e^{C ( 1+ \left| t \right| )^3 } \end{aligned}$$

for initial states of the form $\Psi _N = U_{cN}^* ( \psi ^{\otimes N}\otimes W(\sqrt{N} \varphi )\Omega )$.

Since the quantities $b(\Psi _{N},\varphi )$ and $c(\Psi _{N})$ appearing on the right side of (2.7) and (2.8) are expectation values of unbounded operators, it is not possible to generalize Theorem 2.1 to initial states $\Psi _{N} \notin {\mathcal {D}}( H_N^\mathrm{F})$ via a simple density argument. Using the Gross transform, however, it is possible to obtain a similar result for initial states in a subset of ${\mathcal {D}} ( H_N^0 )$. This follows from Theorem 2.1 in combination with (2.14) and the fact that $U_{K}$ converges strongly to the identity operator for $K\rightarrow \infty$. The precise statement is as follows:

### Theorem 2.2

Let $K_N \geqq c N^{5/6}$ for some $c >0$. Let $(\psi , \varphi ) \in H^2({\mathbb {R}}^3) \times L_1^2({\mathbb {R}}^3)$ with $\left\| \psi \right\| _{L^2({\mathbb {R}}^3)} = 1$, and $\Psi _{N} \in {\mathcal {D}} ( H_{N}^0 )$ such that $\left\| \Psi _{N} \right\| = 1$ and

2.18 $$\begin{aligned} E_0 = \sup _{N \in {\mathbb {N}}} \big \vert N^{-1} \big \langle \Psi _{N} , U_{K_N} H_N^\mathrm{F} U^*_{K_N} \Psi _{N} \big \rangle \big \vert <\infty . \end{aligned}$$

Let $(\psi _t, \varphi _t)$ be the unique solution of (1.6) with initial datum $(\psi , \varphi )$ and $\Psi _{N,t}= e^{-iH_N^\mathrm{F} t}\Psi _{N}$. Then, there exists a constant $C >0$ (depending only on *c*, $\left\| \varphi \right\| _{L_1^2({\mathbb {R}}^3)}$, $\left\| \psi \right\| _{H^2({\mathbb {R}}^3)}$ and $E_0$) such that

2.19 $$\begin{aligned}&\text{ Tr}_{L^2({\mathbb {R}}^3)} \left| \gamma ^{(1,0)}_{\Psi _{N,t}} - | \psi _t \rangle \langle \psi _t | \right| \leqq \sqrt{ a( \Psi _{N},\psi ) + b(\Psi _{N},\varphi ) + c (U_{K_N}^* \Psi _{N}) + N^{-1/2}} e^{C (1+\left| t \right| )^3 } , \end{aligned}$$

2.20 $$\begin{aligned}&\big \langle W^* (\sqrt{N} \varphi _t) \Psi _{N,t} ,\sqrt{ \tfrac{{\mathcal {N}}}{N} } \, W^* (\sqrt{N} \varphi _t) \Psi _{N,t} \big \rangle \nonumber \\ {}&\leqq \sqrt{ a(\Psi _{N},\psi ) + b(\Psi _{N},\varphi ) + c (U_{K_N}^* \Psi _{N}) + N^{-1/2}} e^{C (1+\left| t \right| )^3 } .\end{aligned}$$

In particular, for the Pekar initial state $\Psi _{N} = \psi ^{\otimes N} \otimes W(\sqrt{N} \varphi ) \Omega$ we have the bounds

2.21 $$\begin{aligned}&\text{ Tr}_{L^2({\mathbb {R}}^3)} \left| \gamma ^{(1,0)}_{\Psi _{N,t}} - | \psi _t \rangle \langle \psi _t | \right| \leqq N^{-1/4} e^{C ( 1+ \left| t \right| )^3 } , \end{aligned}$$

2.22 $$\begin{aligned}&\big \langle W^* (\sqrt{N} \varphi _t) \Psi _{N,t} , \sqrt{ \tfrac{{\mathcal {N}}}{N} } \, W^* (\sqrt{N} \varphi _t) \Psi _{N,t} \big \rangle \leqq N^{-1/4} e^{C ( 1+ \left| t \right| )^3 }. \end{aligned}$$

The proof is given in Section 7.3.

### Remark 2.3

The restriction $K_N \geqq c N^{5/6}$ was chosen in order to minimize the error terms in (2.19) and (2.20).

### Remark 2.4

Note that in (2.20) we only control the time evolution of $\sqrt{N^{-1} {\mathcal {N}}}$, while in (2.8) we estimate the operator $N^{-1}{\mathcal {N}}$.

## Preliminaries

### Notation and Basic Estimates

We introduce the usual bosonic creation and annihilation operators

3.1 $$\begin{aligned} a(f)&= \int d^3k \, \overline{f(k)} a_k, \quad a^*(f) = \int d^3k \, f(k) a^*_k , \quad f \in L^2({\mathbb {R}}^3), \end{aligned}$$

as well as the field operators

3.2 $$\begin{aligned} \Phi (f)&= a(f) + a^*(f) , \quad \Pi (f) = \Phi (if) = i \big ( - a(f) + a^*(f) \big ) . \end{aligned}$$

They satisfy the bounds

3.3 $$\begin{aligned} \left\| a(f) \Psi _{N} \right\|&\leqq \left\| f \right\| _{L^2({\mathbb {R}}^3)} \left\| {\mathcal {N}}^{1/2} \Psi _N \right\| , \quad \left\| a^*(f) \Psi _{N} \right\| \leqq \left\| f \right\| _{L^2({\mathbb {R}}^3)} \left\| \left( {\mathcal {N}} + 1 \right) ^{1/2} \Psi _N \right\| , \end{aligned}$$

and

3.4 $$\begin{aligned} \left\| \Phi (f) \Psi _{N} \right\|&\leqq 2 \left\| f \right\| _{L^2({\mathbb {R}}^3)} \left\| \left( {\mathcal {N}} + 1 \right) ^{1/2} \Psi _N \right\| , \quad \nonumber \\ \left\| \Pi (f) \Psi _{N} \right\|&\leqq 2 \left\| f \right\| _{L^2({\mathbb {R}}^3)} \left\| \left( {\mathcal {N}} + 1 \right) ^{1/2} \Psi _N \right\| \end{aligned}$$

for any $\Psi _N\in {\mathcal {H}}^{(N)}$. For $K >0$, we define the classical fields

3.5 $$\begin{aligned} \Phi _{K}(x,t)&= \int _{\left| k \right| \, \leqq K} d^3k \, \left| k \right| ^{-1} \left( e^{ikx} \varphi _t(k) + e^{-ikx} \overline{\varphi _t(k)} \right) , \nonumber \\ \Phi _{\geqq K}(x,t)&= \int _{\left| k \right| \, \geqq K} d^3k \, \left| k \right| ^{-1} \left( e^{ikx} \varphi _t(k) + e^{-ikx} \overline{\varphi _t(k)} \right) .\end{aligned}$$

Moreover, it is useful to define the functions

3.6 $$\begin{aligned} G_{x}(k)&= e^{-i k x} \left| k \right| ^{-1} , \quad \quad G_{K, x}(k) = e^{-i k x} \left| k \right| ^{-1} \mathbb {1}_{\left| k \right| \leqq K}(k), \end{aligned}$$

and $B_{K,x}(k) =\frac{-1}{\left| k \right| (1 + k^2)} e^{-ikx } \mathbb {1}_{\left| k \right| \geqq K}(k)$ as in (2.13). The bounds

3.7 $$\begin{aligned} \left\| G_{K,x} \right\| _{L^2({\mathbb {R}}^3)}^2&= 4 \pi K,\quad \left\| B_{K,x} \right\| _{L^2({\mathbb {R}}^3)}^2 \leqq 4 \pi K^{-3} , \quad \left\| \left| \cdot \right| B_{K,x} \right\| _{L^2({\mathbb {R}}^3)}^2 \leqq 4 \pi K^{-1} \end{aligned}$$

are straightforward to verify and will be frequently used in the rest of the article. We also have

3.8 $$\begin{aligned} \left| \Phi _K(x,t) \right| \leqq \sqrt{32 \pi } \left\| \varphi _t \right\| _{L_1^2({\mathbb {R}}^3) },\quad \left\| \Phi (G_{K,x_j}) \Psi _N \right\|&\leqq \sqrt{16 \pi K} \left\| \left( {\mathcal {N}} + 1 \right) ^{1/2} \Psi _N \right\| \end{aligned}$$

for $$j\in \{1,...,N\}$$.

**Notation:** The functions $k\mapsto k G_{K,x}(k)$ and $k\mapsto k B_{K,x}(k)$ will frequently be denoted by $k G_{K,x}$ and $kB_{K,x}$, respectively. Depending on the context $\left\| \cdot \right\|$ and $\big \langle \cdot , \cdot \big \rangle$ will refer to the norm and scalar product either of ${\mathcal {H}}^{(N)}$ or $L^2({\mathbb {R}}^3)$. If the spaces $L_m^2({\mathbb {R}}^3)$ and $H^m({\mathbb {R}}^3)$ (with $m \in {\mathbb {N}}$) appear as subscripts we will abbreviate them by $L_m^2$ and $H^m$.

### Weyl Operators and Gross Transform

The Weyl operator *W*(*f*) defined in (1.8) is unitary, i.e., $W^*(f) = W^{-1}(f)$, and satisfies the relations

3.9 $$\begin{aligned} W^{-1}(f)\!=\! W(-f), \quad W(f) W(g) \!=\! W(g) W(f) e^{-2 i \mathrm {Im}\langle f , g\rangle } \!=\! W(f+g) e^{-i \mathrm {Im}\langle f , g \rangle } \end{aligned}$$

as well as the shift property

3.10 $$\begin{aligned} W^*(f) a_k W(f) = a_k + f(k). \end{aligned}$$

This immediately implies that the Gross transform, as defined in (2.12), is unitary. Moreover, it has the properties

3.11 $$\begin{aligned} U_K&= W \Big ( - N^{-1/2} \sum _{j=1}^N B_{K,x_j} \Big ) = \prod _{j=1}^N W \Big ( - N^{-1/2} B_{K,x_j} \Big ) \end{aligned}$$

(which holds since $\mathrm {Im}\big \langle B_{K,x} , B_{K,y} \big \rangle =0$ for all $x,y\in {\mathbb {R}}^3$) and

3.12 $$\begin{aligned} U_K a_k U^*_K&= a_k + N^{-1/2} \sum _{j=1}^N B_{K, x_j}(k) . \end{aligned}$$

### The Fröhlich Hamiltonian

In [16], Griesemer and Wünsch give an explicit representation of $H_N^\mathrm{F}$ with the aid of the Gross transform when $N=1$. Below, we state the analogous representation for $N>1$, which will be useful for the proof of our main theorem. Considering $N>1$ does not impose additional difficulties compared to [16].

#### Definition 3.1

With $B_{K,x}$ and $G_{K,x}$ defined in (2.13) and (3.6), respectively, we set

3.13 $$\begin{aligned} A_{K,x}&= - 2 i N^{-1/2} \Big ( \nabla _x \cdot a \big ( k B_{K,x} \big ) + a^* \big ( k B_{K,x} \big ) \cdot \nabla _x \Big ) + N^{-1} \Phi \big ( k B_{K,x} \big )^2, \end{aligned}$$

3.14 $$\begin{aligned} V_{K}(x-y)&= N^{-1} \Big ( \big \langle B_{K,x} , B_{K,y} \big \rangle + 2 \mathrm {Re}\big \langle G_{x} , B_{K,y} \big \rangle \Big ) , \end{aligned}$$

3.15 $$\begin{aligned} H_{N,K}^\mathrm{F}&= \sum _{j=1}^N \left[ - \Delta _j + N^{-1/2} \Phi (G_{K,x_j}) \right] + {\mathcal {N}}, \end{aligned}$$

and define the Gross transformed Fröhlich Hamiltonian as

3.16 $$\begin{aligned} H^\mathrm{G}_{N,K}&= H_{N,K}^\mathrm{F} + \sum _{j=1}^N A_{K,x_j} + \sum _{j,l=1}^N V_K(x_j-x_l). \end{aligned}$$

Note that (3.7) immediately implies the bound

3.17 $$\begin{aligned} \vert V_K(x_j-x_l) \vert \leqq C K^{-1}N^{-1} \end{aligned}$$

for suitable $C>0$. The next result, which is the generalization of [16, Theorem 3.7] to $N\geqq 2$, justifies denoting $H_{N,K}^\mathrm{G}$ as Gross transformed Fröhlich Hamiltonian.

#### Lemma 3.1

The operator $H^\mathrm{G}_{N,K}$ is self-adjoint on ${\mathcal {D}} (H_N^0 )$ for all $K >0$. Moreover, there exists a ${\widetilde{K}}\geqq 0$ such that for all $K \geqq {\widetilde{K}}$ and $N \in {\mathbb {N}}$, the self-adjoint operator $H_N^F$ associated to the quadratic form defined by (1.3) has the representation

3.18 $$\begin{aligned} H_N^\mathrm{F} = U^*_{K} H_{N,K}^\mathrm{G} U_{K}, \quad {\mathcal {D}}(H_N^\mathrm{F}) = U^{*}_{K} {\mathcal {D}}(H_N^0) . \end{aligned}$$

We shall comment on the proof of this lemma in “Appendix A”.

For use below, we also note that there is ${{\widetilde{K}}},C>0$, such that for all $K\geqq {{\widetilde{K}}}$ and $N\geqq 1$,

3.19 $$\begin{aligned} \frac{1}{2} H_{N}^0 - C N&\leqq H_{N}^\mathrm {F} \leqq \frac{3}{2} H_{N}^0 + C N , \end{aligned}$$

3.20 $$\begin{aligned} \frac{1}{2} H_{N}^0 - C N&\leqq H_{N,K}^\mathrm {G} \leqq \frac{3}{2} H_{N}^0 + C N \end{aligned}$$

hold as inequalities on the Hilbert space $L^2({\mathbb {R}}^{3N}) \otimes {\mathcal {F}}_s$ without symmetry constraints on the particles. This will be useful later in order to estimate expectation values with respect to wave functions that are not permutation symmetric in all particle coordinates, as e.g. in (5.8). The derivation of (3.19) and (3.20) is postponed to “Appendix A”.

## Proof of the Main Theorem

We first state three preliminary lemmas from which the proof of Theorem 2.1 then follows easily. The proofs of the lemmas are postponed to later sections.

If we take the limit $K\rightarrow \infty$, the Gross transform has only negligible effect on the one-particle reduced density and the coherent structure of the phonon field. This is quantified in the following lemma, whose proof is given in Sec. 7.1:

### Lemma 4.1

Assume $K \geqq {{\widetilde{K}}}>0$ such that Lemma 3.1 holds. Let $\varphi \in L^2({\mathbb {R}}^3)$, $\Psi _N \in {\mathcal {D}}((H_N^\mathrm{F})^{1/2})$ with $\left\| \Psi _N \right\| =1$, and the Gross transform $U_{K}$ defined as in (2.12). Then,

4.1 $$\begin{aligned} \text{ Tr}_{L^2({\mathbb {R}}^3)} \Big \vert \gamma ^{(1,0)}_{\Psi _N} - \gamma ^{(1,0)}_{U_{K} \Psi _N} \Big \vert&\leqq \frac{C}{K^{3/2}} \left\| \Big ( \frac{H_N^\mathrm {F} + C N}{N}\Big )^{1/2} \Psi _N \right\| \end{aligned}$$

and

4.2 $$\begin{aligned} N^{-1}&\left| \big \langle W^*(\sqrt{N} \varphi ) \Psi _{N} , \big ( {\mathcal {N}} - U_K^* {\mathcal {N}} U_K \big ) W^* (\sqrt{N} \varphi ) \Psi _N \big \rangle \right| \nonumber \\ {}&\quad \le \frac{C (1+ \left\| \varphi \right\| _2)}{K^{3/2}} \left\| \Big ( \frac{H_N^\mathrm {F} + C N}{N}\Big )^{1/2} \Psi _N \right\| \end{aligned}$$

for some $$C>0$$.

Next, we define a functional to compare $U_{K}\Psi _{N,t}$ with the Pekar state $\psi _t^{\otimes N}\otimes W(\sqrt{N} \varphi _t)\Omega$. To this end, we introduce for $j \in \{1,...,N\}$ the projections $p^\psi _j : L^2({\mathbb {R}}^{3N}) \rightarrow L^2({\mathbb {R}}^{3N})$ and $q_j^{\psi } : L^2({\mathbb {R}}^{3N}) \rightarrow L^2({\mathbb {R}}^{3N})$, given by

4.3 $$\begin{aligned} p_j^{\psi } f_N(x_1, \ldots , x_N)&= \psi (x_j) \int d^3 x_j' \, \overline{ \psi (x_j')} f_N(x_1,\ldots x_{j-1},x_j',x_{j+1},\ldots , x_N) \end{aligned}$$

for $f_N \in L^2({\mathbb {R}}^{3N})$, and $q_j^{\psi } = 1 - p_j^{\psi }$. (More compactly, in bracket notation, $p^\psi _j = \vert \psi \rangle \langle \psi \vert _j$).

### Definition 4.1

Let $K>0$ and $\left( \psi , \varphi \right) \in L^2({\mathbb {R}}^3) \times L^2({\mathbb {R}}^3)$ with $\left\| \psi \right\| =1$ and $\Psi _{N}\in {\mathcal {D}} \left( H_N^\mathrm{F} \right)$, $\left\| \Psi _{N} \right\| = 1$. We define $\beta ^a_K : {\mathcal {D}} ( H_N^\mathrm{F} ) \times L^2({\mathbb {R}}^3) \rightarrow {\mathbb {R}}_0^+$, $\beta ^b_K: {\mathcal {D}} ( H_N^\mathrm{F} ) \times L^2({\mathbb {R}}^3) \rightarrow {\mathbb {R}}_0^+$ and $\beta ^c: {\mathcal {D}} ( H_N^\mathrm{F} ) \rightarrow {\mathbb {R}}_0^+$ by

4.4 $$\begin{aligned} \beta ^a_K \left( \Psi _{N }, \psi \right)&= \big \langle \Psi _{N } , U^*_K \left( q_1^{\psi } \otimes \mathbb {1}_{{\mathcal {F}}_s} \right) U_K \Psi _{N } \big \rangle , \end{aligned}$$

4.5 $$\begin{aligned} \beta ^b_{K} \left( \Psi _{N }, \varphi \right)&= N^{-1} \big \langle W^*(\sqrt{N} \varphi ) U_K \Psi _{N} , {\mathcal {N}} W^*(\sqrt{N} \varphi ) U_K \Psi _N \big \rangle \end{aligned}$$

4.6 $$\begin{aligned} \beta ^c(\Psi _{N })&= \left\| N^{-1} \left( H_N^\mathrm{F} - \big \langle \Psi _{N } , H_N^\mathrm{F} \Psi _{N } \big \rangle \right) \Psi _{N } \right\| ^2. \end{aligned}$$

Moreover, we define $\beta _{K} : {\mathcal {D}} \left( H_N^\mathrm{F} \right) \times L^2({\mathbb {R}}^3) \times L^2({\mathbb {R}}^3) \rightarrow {\mathbb {R}}_0^+$ by

4.7 $$\begin{aligned} \beta _K(\Psi _N,\psi ,\varphi )&= \beta ^a_K(\Psi _N,\psi ) + \beta ^b_K(\Psi _N,\varphi ) + \beta ^c(\Psi _N) . \end{aligned}$$

For solutions $\Psi _{N,t}$ and $(\psi _t,\varphi _t)$ of the Schrödinger equation (1.2) and the Landau–Pekar equations (1.6), respectively, we use the shorthand notations

$$\begin{aligned} \beta _K(t)&= \beta _K(\Psi _{N,t},\psi _t,\varphi _t),\ \ \beta ^a_K(t) = \beta ^a_K (\Psi _{N,t},\psi _t), \ \ \\ \beta ^b_K(t)&= \beta ^b_K (\Psi _{N,t},\varphi _t), \ \ \beta ^c(t) = \beta ^c (\Psi _{N,t}) . \end{aligned}$$

### Remark 4.1

Note that

4.8 $$\begin{aligned} \beta ^b_K \left( \Psi _{N }, \varphi \right) = \int d^3k \, \left\| \left( N^{-1/2} a_k - \varphi (k) \right) U_K \Psi _{N } \right\| ^2. \end{aligned}$$

### Remark 4.2

$\beta _K(t)$ being small compared to one ensures that

- the *N*-particle component of $U_K \Psi _{N,t}$ is approximately given by the product $\psi _t^{\otimes N}$—more precisely, $\beta ^a_K(t)$ measures the relative number of particles not in $\psi _t$,
- the phonon component of $U_K \Psi _{N,t}$ is close to the coherent state $W(\sqrt{N} \varphi _t)\Omega$—more, precisely, $\beta ^b_K(t)$ measures the relative number of excitations with respect to the coherent state $W(\sqrt{N} \varphi _t)\Omega$,
- the variance of $N^{-1} H_N^\mathrm{F}$ with respect to $\Psi _{N,t}$ is small compared to one—this will be used to control the singular ultraviolet behavior of the phonon field (for a detailed explanation of this point, see the beginning of Section 5). Also note that $\beta ^c (\Psi _{N,t}) = \beta ^c(\Psi _{N})$ is a conserved quantity, and thus requiring $\beta ^c$ to be small only poses a restriction on the initial state. Since $\beta ^c(\Psi _{N}) = c(\Psi _N)$, Proposition 2.2 shows that $\beta ^c$ is small for initial states of the form $\Psi _N = U_K^* \psi ^{\otimes N}\otimes W(\sqrt{N} \varphi )\Omega$ with $K=K_N$ large enough.

The functional $\beta _K(t)$ can consequently be used to monitor whether the condensate of the particles and the coherent state of the phonons is stable during the time evolution. Its definition is motivated by a previous work on the derivation of the Maxwell–Schrödinger equations [23]. In addition it is necessary to include the Gross transform in the definition of $\beta ^a_K(t)$ and $\beta ^b_K(t)$. This induces correlations between the electron and the phonons and effectively regularizes the interaction. In this sense, the Gross transform has a similar role as the Bogoliubov transformation in the derivation of the time-dependent Gross-Pitaevskii equation (see for instance [2, 3, 20, 30]).

The trace norm of the difference $\gamma ^{(1,0)}_{U_K \Psi _{N,t}} - \vert \psi _t \rangle \langle \psi _t \vert$ and the quantity $\beta ^a_K(t)$ are related by

4.9 $$\begin{aligned} \beta ^a_K (t)&\leqq \text {Tr}_{L^2({\mathbb {R}}^3)} \left| \gamma _{U_K \Psi _{N,t}}^{(1,0)} - | \psi _t \rangle \langle \psi _t | \right| \leqq 4 \sqrt{\beta ^a_K(t)} , \end{aligned}$$

which is the content of the following lemma when $\Psi _N = U_K \Psi _{N,t}$:

### Lemma 4.2

Let $\psi \in L^2({\mathbb {R}}^3)$ with $\left\| \psi \right\| =1$ and $\Psi _{N} \in {\mathcal {H}}^{(N)}$ with $\left\| \Psi _N \right\| =1$. Then,

4.10 $$\begin{aligned} \big \langle \Psi _{N } , \big ( q_1^{\psi } \otimes \mathbb {1}_{{\mathcal {F}}_s} \big ) \Psi _{N } \big \rangle&\leqq \text {Tr}_{L^2({\mathbb {R}}^3)} \left| \gamma _{\Psi _{N}}^{(1,0)} - | \psi \rangle \langle \psi | \right| \leqq 4 \sqrt{\big \langle \Psi _{N } , \big ( q_1^{\psi } \otimes \mathbb {1}_{{\mathcal {F}}_s} \big ) \Psi _{N } \big \rangle } . \end{aligned}$$

### Proof

The lemma is a consequence of the identity

4.11 $$\begin{aligned} \text {Tr}_{L^2({\mathbb {R}}^3)} \left| \gamma _{\Psi _{N}}^{(1,0)} - | \psi \rangle \langle \psi | \right| = \sup _{\left\| A \right\| _{op}=1} \left| \big \langle \Psi _N , A_{1} \Psi _N \big \rangle - \big \langle \psi , A \psi \big \rangle \right| , \end{aligned}$$

where the supremum is taken over all bounded operators $A:L^2({\mathbb {R}}^3) \rightarrow L^2({\mathbb {R}}^3)$ and

4.12 $$\begin{aligned} A_1 = \big ( A \otimes \underbrace{\mathbb {1}_{L^2({\mathbb {R}}^3)} \otimes ... \otimes \mathbb {1}_{L^2({\mathbb {R}}^3)}}_{ N -1\ \text {times}} \big ) \otimes \mathbb {1}_{{\mathcal {F}}_s} \end{aligned}$$

acts non-trivially only on the variable $x_1$. (Note that (4.11) holds because the space of bounded operators is the dual of the space of trace-class operators). The first bound then follows from

4.13 $$\begin{aligned} \big \langle \Psi _{N } , \big ( q_1^{\psi } \otimes \mathbb {1}_{{\mathcal {F}}_s} \big ) \Psi _{N } \big \rangle = \big \langle \Psi _N , q_1^\psi \Psi _N \big \rangle = \left| \big \langle \Psi _N , p_1^\psi \Psi _N \big \rangle - \big \langle \psi , p^\psi \psi \big \rangle \right| , \end{aligned}$$

while for the second bound, one inserts $1=p_1^\psi +q_1^\psi$ on the left and right of $A_{1}$ and uses

4.14 $$\begin{aligned} p_1^\psi A_{1} p_1^\psi - \big \langle \psi , A \psi \big \rangle = q_1^\psi \big \langle \psi , A \psi \big \rangle \end{aligned}$$

together with the Cauchy–Schwarz inequality for the remaining terms.^3^$\square$

The main ingredient of the proof of Theorem 2.1 is the following estimate for $\beta _K(t)$:

### Lemma 4.3

Assume $$K \geqq {{\widetilde{K}}}>0$$ such that Lemma 3.1 holds. Let $$\Psi _{N,t} = e^{-iH_N^\mathrm{F} t }\Psi _{N} $$ with $$\Psi _{N} \in {\mathcal {D}} ( H_N^\mathrm{F} )$$ such that $$\left\| \Psi _{N} \right\| = 1$$ and $$E_0 = \sup _{N \in {\mathbb {N}}} \left| N^{-1} \big \langle \Psi _{N} , H_N^\mathrm{F} \Psi _{N} \big \rangle \right| <\infty $$. Let further $$(\psi _t, \varphi _t)$$ be a solution of (1.6) with $$(\psi , \varphi ) \in H^2({\mathbb {R}}^3) \times L^2_{1}({{\mathbb {R}}^3})$$ such that $$\left\| \psi \right\| = 1$$. Then, there exists a constant $$C > 0 $$ only depending on $$\left\| \varphi \right\| _{L_1^2 }$$, $$\left\| \psi \right\| _{H^2}$$, and $$E_0$$, such that

4.15 $$\begin{aligned} \left| \frac{\mathrm{d}}{\mathrm{d}t} \beta _K(t) \right|&\leqq C \left( 1 + t^2 \right) \left( \beta _K(t ) + K N^{-1} + K^{-1} \right) . \end{aligned}$$

The proof is given in Section 6. Putting the above statements together, we obtain the proof of Theorem 2.1.

### Proof of Theorem 2.1

We first apply Grönwall’s argument to (4.15) in order to obtain

4.16 $$\begin{aligned} \beta _K(t )&\leqq e^{C ( 1+ \left| t \right| )^3} \left( \beta _K(0 ) + K N^{-1} + K^{-1} \right) . \end{aligned}$$

Next, set $K = K_N = {{\widetilde{K}}}N^{1/2}$ with ${{\widetilde{K}}}>0$ as in Lemma 3.1, and compute

4.17 $$\begin{aligned} \text{ Tr}_{L^2({\mathbb {R}}^3)} \Big \vert \gamma ^{(1,0)}_{\Psi _{N,t}} - \vert \psi _t \rangle \langle \psi _t \vert \Big \vert&\leqq \text{ Tr}_{L^2({\mathbb {R}}^3)} \Big \vert \gamma ^{(1,0)}_{U_{K_N}\Psi _{N,t}} - \vert \psi _t \rangle \langle \psi _t \vert \Big \vert + CN^{-3/4} \nonumber \\ {}&\leqq 4 \sqrt{\beta ^a_{K_N}(t)} + C N^{-3/4} \nonumber \\ {}&\leqq 4 \sqrt{ \beta _{K_N}(t)} + C N^{-3/4} \nonumber \\ {}&\leqq \sqrt{\beta _{K_N}(0) + N^{-1/2}} e^{C(1+ \left| t \right| )^3}, \end{aligned}$$

where we used inequality (4.1) in the first step, Lemma (4.2) in the second and (4.16) in the last one. The estimate (2.7) then follows from $\beta ^c(0) = c(\Psi _{N})$ and

4.18 $$\begin{aligned} \beta ^a_{K_N}(0) + \beta ^b_{K_N}(0) \leqq a(\Psi _{N},\psi ) + b(\Psi _{N},\varphi ) + C N^{-3/4}, \end{aligned}$$

which in turn holds because of (4.9) and Lemma 4.1.

Using (4.2), we can similarly estimate

4.19 $$\begin{aligned}&N^{-1} \big \langle W^*(\sqrt{N} \varphi _t) \Psi _{N,t} , {\mathcal {N}} W^*(\sqrt{N} \varphi _t) \Psi _{N,t} \big \rangle \nonumber \\ {}&\quad \leqq N^{-1} \big \langle W^*(\sqrt{N} \varphi _t) U_{K_N} \Psi _{N,t} , {\mathcal {N}} W^*(\sqrt{N} \varphi _t) U_{K_N} \Psi _{N,t} \big \rangle + C (1+\left\| \varphi _t \right\| ) N^{-3/4} \nonumber \\ {}&\quad = \beta ^b_{K_N}(t) + C (1+\left\| \varphi _t \right\| ) N^{-3/4} \nonumber \\ {}&\quad \leqq \big ( \beta _{K_N}(0) + N^{-1/2}\big ) e^{C( 1 + \left| t \right| )^3} \nonumber \\ {}&\quad \leqq \big ( a(\Psi _{N},\psi ) + b(\Psi _{N},\varphi ) + c(\Psi _{N}) + N^{-1/2} \big ) e^{C( 1+ \left| t \right| )^3}. \end{aligned}$$

This completes the proof of the theorem. $\square$

The proofs of Proposition 2.2 and Theorem 2.2 are postponed to Sections 7.2 and 7.3, respectively.

## Bound on $\Vert \nabla _2 q_1^{\psi } U_{K} \Psi _{N}\Vert$

In this section, we state and prove a bound that is a crucial ingredient in the proof of Lemma 4.3.

### Lemma 5.1

Assume $K\geqq {{\widetilde{K}}}>0$ such that Lemma 3.1 holds. Let $(\psi , \varphi ) \in H^2({\mathbb {R}}^3) \times L_1^2({\mathbb {R}}^3)$ and $\Psi _{N} \in {\mathcal {D}} ( H_N^\mathrm{F} )$ with $\left\| \Psi _N \right\| =1$, and set $E_N^\mathrm{F}(\Psi _N) = N^{-1} \big \langle \Psi _N , H_N^\mathrm{F}\Psi _N \big \rangle$. Then

5.1 $$\begin{aligned} \left\| \nabla _2 q_1^{\psi } U_{K} \Psi _{N} \right\| ^2&\leqq g(\Psi _N,\psi ,\varphi ) \left( \beta _K(\Psi _N,\psi ,\varphi ) + N^{-1} K^{-1} + N^{-2} K \right) , \end{aligned}$$

where $g(\Psi _N,\psi ,\varphi ) = C (\left\| \psi \right\| _{H^2}^2 + \left\| \varphi \right\| _{L_1^2}^2 + \vert E_N^\mathrm{F}(\Psi _N) \vert )$ for some $C>0$.

Before we give its proof, we explain the importance of the above estimate. The main technical difficulty for controlling the time-derivative of $\beta _K(t)$ arises from the singular ultraviolet behavior of the phonon field. In particular, if we want to estimate $\frac{\mathrm{d}}{\mathrm{d}t}\beta ^b_K(t)$, we have to bound the following term (cf. Section 6.2)

5.2 $$\begin{aligned} (6.21{\mathrm{d}})&= \!- 2 \mathrm {Im}\big \langle U_{K} \Psi _{N,t} , \int _{\left| k \right| \leqq K}\! d^3k \, \left| k \right| ^{-1} e^{ikx_1} q_1^{\psi _t} \left( N^{-1/2} a_k - \varphi _t(k) \right) U_{K} \Psi _{N,t} \big \rangle \end{aligned}$$

by an *N*-independent constant times the functional $\beta _K(t)$. A naive estimate using the Cauchy–Schwarz inequality would give the bound

5.3 $$\begin{aligned} \left| (6.21\mathrm{d}) \right|&\leqq C K^{1/2} \sqrt{\beta ^a_{K}(t) \beta ^b_{K}(t) }, \end{aligned}$$

which is not sufficient for $K \gg 1$. The reason for the bad behavior for large *K* clearly comes from the careless estimate of the form factor $\vert k \vert ^{-1}$.

The most obvious strategy for a better estimate is to apply the well-known commutator method of Lieb and Yamazaki [26], which utilizes the particle momentum in order to obtain a better ultraviolet behavior of the phonon field. More precisely, one writes the exponential $e^{ik x_1}$ in terms of a commutator with the gradient $i\nabla _{x_1}$,

5.4 $$\begin{aligned} e^{ik x_1} = \big ( 1 + \left| k \right| ^{2} \big )^{-1} \left( e^{ik x_1} - k \cdot \big [ i \nabla _{x_1}, e^{ik x_1} \big ] \right) , \end{aligned}$$

which suggests a better decay for large $\vert k \vert$ provided that one has some control of the regularity of the particle with coordinate $x_1$. Using this identity together with $p_1^{\psi _t} + q_1^{\psi _t} =1$, we find by a straightforward computation that (6.21d) can be written as

5.5 $$\begin{aligned} - 2 \mathrm {Im}\int _{\left| k \right| \leqq K} d^3k \,&\left| k \right| ^{-1} \big ( 1 + \left| k \right| ^{2} \big )^{-1} \nonumber \\ {}&\; \times \bigg ( \big \langle e^{-ikx_1} \left( N^{-1/2} a_k^* - \overline{\varphi _t(k)} \right) U_{K} \Psi _{N,t} , q_1^{\psi _t} U_{K} \Psi _{N,t} \big \rangle \nonumber \\ {}&\quad \; - \big \langle e^{-ikx_1} \left( N^{-1/2} a_k^* - \overline{\varphi _t(k)} \right) k \cdot i \nabla _1 p_1^{\psi _t} U_{K} \Psi _{N,t} , q_1^{\psi _t} U_{K} \Psi _{N,t} \big \rangle \nonumber \\ {}&\quad \; - \big \langle e^{-ikx_1} k \cdot i \nabla _1 q_1^{\psi _t} U_{K} \Psi _{N,t} , \left( N^{-1/2} a_k - \varphi _t(k) \right) q_1^{\psi _t} U_{K} \Psi _{N,t} \big \rangle \nonumber \\ {}&\quad \; + \big \langle e^{-ikx_1} \left( N^{-1/2} a_k^* - \overline{\varphi _t(k)} \right) U_{K} \Psi _{N,t} , k \cdot i \nabla _1 q_1^{\psi _t} U_{K} \Psi _{N,t} \big \rangle \bigg ). \end{aligned}$$

With the aid of the Cauchy–Schwarz inequality and the canonical commutation relations this implies the bound

5.6 $$\begin{aligned} \left| (6.21\mathrm{d}) \right|&\leqq C\left( \left\| \psi _t \right\| _{H^1} \sqrt{\beta ^a_K(t)} + \left\| \nabla _1 q_1^{\psi _t} U_{K} \Psi _{N,t} \right\| \right) \sqrt{\beta ^b_K(t) + N^{-1}}. \end{aligned}$$

Contrary to (5.3), there is no more divergence for large *K*. However, the above inequality contains the new term $\Vert \nabla _1 q_1^{\psi _t} U_{K} \Psi _{N,t} \Vert$. Thus if we want to apply Grönwall’s inequality we would have to show that this term is small compared to one or bounded by a constant times $\sqrt{\beta _{K}(t)}$.^4^ It is not clear how to derive such a bound, however, and hence, we are forced to estimate $\left| (6.21\mathrm{d}) \right|$ in a different way.

A possible solution to this problem is to use a combination of the estimates from [23, Chapter VIII.1] with an operator bound that is motivated by [10, Lemma 10] (see Section 6 for the detailed argument). In short, we use the symmetry of the wave function and an estimate that is similar in spirit to the commutator method of Lieb and Yamazaki to obtain

5.7 $$\begin{aligned} \left| (6.21\mathrm{d}) \right|&\leqq C \left( \beta ^a_{K}(t) + \beta ^b_{K}(t) + N^{-1} K + \left\| \nabla _2 q_1^{\psi _t} U_{K} \Psi _{N,t} \right\| ^2 \right) . \end{aligned}$$

As shown in Lemma 5.1, the new quantity $\Vert \nabla _2 q_1^{\psi _t} U_{K} \Psi _{N,t} \Vert ^2$ can be bounded by $\beta _{K}(t)$ and errors proportional to $N^{-1}K^{-1}$ and $N^{-2}K$.

### Proof of Lemma 5.1

Using the symmetry of $\Psi _{N}$ and $- \Delta _1 \geqq 0$, we can bound

5.8 $$\begin{aligned} \left\| \nabla _2 q^\psi _1 U_K \Psi _{N} \right\| ^2&= (N-1)^{-1} \sum _{j=2}^N \big \langle q_1^\psi U_K \Psi _{N} , (-\Delta _j)q_1^\psi U_K \Psi _{N} \big \rangle \nonumber \\&\leqq \frac{2}{N} \sum _{j=1}^N \big \langle q_1^\psi U_K \Psi _{N} , (-\Delta _j)q_1^\psi U_K \Psi _{N} \big \rangle . \end{aligned}$$

With $-\sum _{j=1}^N \Delta _{j} \leqq H_N^0$ and (3.20), we thus have

5.9 $$\begin{aligned} \left\| \nabla _2 q_1^\psi U_K \Psi _{N} \right\| ^2 \leqq C \beta ^a_K(\Psi _N,\psi ) + 4 N^{-1} \big \langle q_1^\psi U_K \Psi _{N} , H_{N,K}^\mathrm {G} q_1^\psi U_K \Psi _{N} \big \rangle . \end{aligned}$$

By using $q_1^\psi H_{N,K }^\mathrm{G} q_1^\psi = q_1^\psi H_{N,K }^\mathrm{G} - q_1^\psi H_{N,K}^\mathrm{{G}} p_1^\psi$ and recalling Definition (3.1), we get

5.10a $$\begin{aligned} \left\| \nabla _2 q_1^\psi U_K \Psi _{N} \right\| ^2&\leqq C \Big ( \beta ^a_K(\Psi _N,\psi ) + N^{-1} \left| \big \langle q_1^\psi U_K \Psi _{N} , H_{N,K}^\mathrm{G} U_K \Psi _{N} \big \rangle \right| \end{aligned}$$

5.10b $$\begin{aligned}&\quad + N^{-1} \left| \big \langle U_K \Psi _{N} , q_1^\psi \left( - \Delta _1 \right) p_1^\psi U_K \Psi _{N} \big \rangle \right| \end{aligned}$$

5.10c $$\begin{aligned}&\quad + N^{-3/2} \left| \big \langle U_K \Psi _{N} , q_1^\psi \Phi (G_{K,x_1}) p_1^\psi U_K \Psi _{N} \big \rangle \right| \end{aligned}$$

5.10d $$\begin{aligned}&\quad + N^{-1} \left| \big \langle U_K \Psi _{N} , q_1^\psi A_{K ,x_1} p_1^\psi U_K \Psi _{N} \big \rangle \right| \end{aligned}$$

5.10e $$\begin{aligned}&\quad + \left| \big \langle U_K \Psi _{N} , q_1^\psi V_K(x_1-x_2) p_1^\psi U_K \Psi _{N} \big \rangle \right| \Big ) . \end{aligned}$$

In what follows, we shall bound the various terms on the right hand side.

**Line** (5.10a). In the second summand in this line, we add and subtract $E_N^\mathrm{F}(\Psi _N) \beta ^a_K(\Psi _N,\varphi )$, to obtain

5.11 $$\begin{aligned}&N^{-1}\left| \big \langle q_1^\psi U_K \Psi _{N} , H_{N,K}^\mathrm {G} U_K \Psi _{N} \big \rangle \right| \nonumber \\ {}&\quad \leqq \left| \big \langle q_1^\psi U_K \Psi _{N} , \left( N^{-1} H_{N,K}^\mathrm {G} - E_N^\mathrm {F}(\Psi _N) \right) U_K \Psi _{N} \big \rangle \right| + E_N^\mathrm {F}(\Psi _N) \beta ^a_K(\Psi _N,\psi ). \end{aligned}$$

With the aid of the Cauchy–Schwarz inequality and (3.18), we find

5.12 $$\begin{aligned} \left| (5.10\mathrm{a}) \right| \leqq C (1+ \vert E_N^\mathrm{F}(\Psi _N) \vert ) \left( \beta _K^a(\Psi _N,\psi ) + \beta ^c(\Psi _N) \right) . \end{aligned}$$

**Line** (5.10b). One readily obtains

5.13 $$\begin{aligned} (5.10\mathrm{b})&\leqq N^{-1} \left\| \left( - \Delta _1 \right) p_1^\psi U_K \Psi _{N} \right\| \left\| q_1^\psi U_K \Psi _{N} \right\| \nonumber \\&\leqq \frac{1}{2} \left\| \psi \right\| _{H^2} \left( \beta ^a_K(\Psi _N,\psi ) + N^{-2} \right) . \end{aligned}$$

**Line** (5.10c). Using (3.8), we find

5.14 $$\begin{aligned} (5.10\mathrm{c})&\leqq N^{-3/2} \left\| q_1^\psi U_K \Psi _{N} \right\| \left\| \Phi \left( G_{K,x_1} \right) p_1^\psi U_K \Psi _{N} \right\| \nonumber \\&\leqq C N^{-3/2} K^{1/2} \sqrt{\beta ^a_K(\Psi _N,\psi )\, \big \langle U_K \Psi _{N} , ( {\mathcal {N}} +1) U_K \Psi _{N} \big \rangle } \end{aligned}$$

and hence, using (3.20), (3.18) and ${\mathcal {N}} \leqq H_N^0$, we have

5.15 $$\begin{aligned} (5.10\mathrm{c})&\leqq C (1 + \vert E_N^\mathrm{F}(\Psi _N) \vert ) \left( KN^{-2} + \beta ^a_K(\Psi _N,\psi ) \right) . \end{aligned}$$

**Line** (5.10d). We recall the definition of $A_{K,x}$ in (3.13) and estimate the term with $a^*(kB_{K,x}) \cdot \nabla _x$ by

5.16 $$\begin{aligned}&\left| \big \langle U_K \Psi _{N} , q_1^\psi N^{-1/2} a^*(kB_{K,x_1}) \cdot \nabla _1 p_1^\psi U_K \Psi _{N} \big \rangle \right| \nonumber \\ {}&\quad \leqq \int d^3k \ \left| \big \langle q_1^\psi \Big ( N^{-1/2} a_k - \varphi (k) + \varphi (k) \Big ) U_K \Psi _{N} , k B_{K,x_1}(k) \cdot \nabla _1 p_1^\psi U_K \Psi _{N} \big \rangle \right| \nonumber \\ {}&\quad \leqq \left\| \nabla _1 p_1^\psi U_K \Psi _{N} \right\| \int d^3k \left( \left| k B_{K,x} (k) \right| \left\| \Big ( N^{-1/2} a_k - \varphi (k) \Big ) U_K \Psi _{N} \right\| + \left| k B_{K,x} (k) \varphi (k) \right| \right) \nonumber \\ {}&\quad \leqq \left\| \psi \right\| _{H^1 } \left( \left\| \left| \cdot \right| B_{K,x} \right\| \, \sqrt{\beta _K^b(\Psi _N,\varphi ) } + \left\| B_{K,x} \right\| \, \left\| \left| \cdot \right| \varphi \right\| \right) \nonumber \\ {}&\quad \leqq C \left\| \psi \right\| _{H^1 } \left( K^{-1/2} \sqrt{ \beta ^b_K(\Psi _N,\varphi )} + K^{-3/2} \left\| \varphi \right\| _{L_1^2} \right) . \end{aligned}$$

Using $q_1^\psi = 1-p_1^\psi$ and $-\Delta _1\leqq N^{-1} H_N^0$ as quadratic forms on $L^2_s({\mathbb {R}}^{3N}) \otimes {\mathcal {F}}_s$, together with (3.20), we find

5.17 $$\begin{aligned} \left\| \nabla _1 q_1^\psi U_K \Psi _{N} \right\| ^2 \leqq 2 \left( \left\| \nabla _1 p_1^\psi U_K \Psi _{N} \right\| ^2 + \left\| \nabla _1 U_K \Psi _{N} \right\| ^2\right) \leqq C \left( \left\| \psi \right\| _{H^1 }^2 + \vert E_N^\mathrm {F} (\Psi _N) \vert + 1\right) . \end{aligned}$$

With this at hand, we can proceed for the term with $\nabla _x \cdot a(kB_{K,x})$ similarly as in (5.16), with the result that

5.18 $$\begin{aligned}&\left| \big \langle U_K \Psi _{N} , q_1^\psi \nabla _1 \cdot N^{-1/2} a(kB_{K,x_1}) p_1^\psi U_K \Psi _{N} \big \rangle \right| \nonumber \\ {}&\quad \leqq \left\| \nabla _1 q_1^\psi U_K \Psi _N \right\| \int d^3k \left( \left| kB_{K,x}(k) \right| \left\| \Big ( N^{-1/2}a_k - {\varphi (k)} \Big ) U_K \Psi _{N} \right\| + \left| k B_{K,x}(k) \varphi (k) \right| \right) \nonumber \\ {}&\quad \leqq C \sqrt{\left\| \psi \right\| _{H^1 }^2 + \vert E_N^\mathrm {F} (\Psi _N) \vert } \left( K^{-1/2} \sqrt{ \beta ^b_K(\Psi _N,\varphi )} + K^{-3/2} \left\| \varphi \right\| _{L_1^2} \right) . \end{aligned}$$

Next, we estimate the term in line (5.10d) with $\Phi (kB_{K,x})^2$,

5.19 $$\begin{aligned}&\left| \big \langle U_K \Psi _{N} , q_1^\psi N^{-1} \Phi ( kB_{K,x_1} )^2 p_1^\psi U_K \Psi _{N} \big \rangle \right| \nonumber \\ {}&\quad \leqq N^{-1} \left\| \Phi ( kB_{K,x_1} ) q_1^\psi U_K\Psi _{N} \right\| \, \left\| \Phi ( kB_{K,x_1} ) p_1^\psi U_K \Psi _{N} \right\| \nonumber \\ {}&\quad \leqq C N^{-1} \left\| \left| \cdot \right| B_{K ,x} \right\| ^2 \big \langle U_K \Psi _{N} , ({\mathcal {N}}+1) U_K \Psi _N \big \rangle \nonumber \\ {}&\quad \leqq C K^{-1} N^{-1} \Big ( \big \langle U_K \Psi _{N} , ( H_{N,K }^\mathrm {G} +C N ) U_K \Psi _{N} \big \rangle + 1 \Big ) \nonumber \\ {}&\quad \leqq C \left( \vert E_N^\mathrm {F}(\Psi _N) \vert + 1 \right) K^{-1} . \end{aligned}$$

By summing up the terms, we obtain the bound

5.20 $$\begin{aligned}&\left| \big \langle U_K \Psi _N , q_1^\psi A_{K,x_1} p_1^\psi U_K \Psi _N \big \rangle \right| \nonumber \\ {}&\quad \leqq C \big (\left\| \psi \right\| _{H^1}^2 + \left\| \varphi \right\| _{L_1^2}^2 + \vert E_N^\mathrm {F}(\Psi _N) \vert \big ) \left( K^{-1} + \beta _K^b(\Psi _N,\varphi ) \right) . \end{aligned}$$

**Line** (5.10e). Using (3.17),

5.21 $$\begin{aligned} \left| \big \langle U_K\Psi _{N} , q_1^\psi V_K(x_1-x_2) p_1^\psi U_K \Psi _{N} \big \rangle \right|&\leqq \sqrt{ \beta ^a_K(\Psi _N, \psi ) } \left\| V_K(x_1-x_2) p_1^\psi U_K \Psi _{N} \right\| \nonumber \\ {}&\leqq C\big ( \beta ^a_K(\Psi _N,\psi ) + N^{-2} K^{-2}\big ) . \end{aligned}$$

This completes the proof of the lemma. $\square$

## Proof of Lemma 4.3 (Time Derivative of $\beta _K(t)$)

We first observe that

6.1 $$\begin{aligned} \frac{\mathrm{d}}{\mathrm{d}t} U_{K} \Psi _{N,t}&= - i U_{K} H^\mathrm{F}_N \Psi _{N,t} = - i U_{K} H^\mathrm{F}_N U_{K}^* U_{K} \Psi _{N,t} = - i H_{N,K}^\mathrm{G} U_{K} \Psi _{N,t}, \end{aligned}$$

from which it follows readily that $\frac{\mathrm{d}}{\mathrm{d}t}\beta ^c(t) = 0$. The time-derivatives of $\beta ^a_K(t)$ and $\beta ^b_K(t)$ are estimated in the next two sections. Throughout both sections, we use the abbreviation $E_N^\mathrm{F}(\Psi _{N}) =N^{-1} \big \langle \Psi _N , H_N^\mathrm{F}\Psi _N \big \rangle$.

### Time Derivative of $\beta ^a_K(t)$

For $q_1^t= q_1^{\psi _t} = 1 - p_1^{\psi _t}$, we have

6.2 $$\begin{aligned} \frac{\mathrm{d}}{\mathrm{d}t} q_1^{t} = - \frac{\mathrm{d}}{\mathrm{d}t}p_1^{t} = i \big [-\Delta _1 + \Phi (x_1,t) , p_1^{t}\big ] = - i \big [-\Delta _1 + \Phi (x_1 ,t) , q_1^{t}\big ] . \end{aligned}$$

Using this together with (6.1), we compute

6.3 $$\begin{aligned} \frac{\mathrm{d}}{\mathrm{d}t}\beta ^a_K (t)&= \frac{\mathrm{d}}{\mathrm{d}t} \big \langle U_K \Psi _{N,t} , q_1^t U_K\Psi _{N,t} \big \rangle \nonumber \\&= - 2\mathrm {Im}\big \langle U_K \Psi _{N,t} , \left( H_{N,K}^\mathrm{G} + \Delta _1 - \Phi (x_1,t) \right) q_1^t U_K \Psi _{N,t} \big \rangle \nonumber \\&= - 2\mathrm {Im}\big \langle U_K \Psi _{N,t} , p_1^t\left( H_{N,K}^\mathrm{G} + \Delta _1 - \Phi (x_1,t) \right) q_1^t U_K \Psi _{N,t} \big \rangle , \end{aligned}$$

where we inserted $1= p_1^t+q_1^t$ and used that the term with $q_1^t$ on both sides is real. Recall Definition 3.1. Using $\Phi (x_1,t) = \Phi _{K}(x_1,t) + \Phi _{\geqq K}(x_1,t)$, $p_1^tq_1^t = 0$ and the symmetry of $\Psi _N$, we can rewrite (6.3) as

6.4a $$\begin{aligned} \frac{\mathrm{d}}{\mathrm{d}t}\beta ^a_K (t)&= - 2\mathrm {Im}\big \langle U_K \Psi _{N,t} , p_1^t\left( N^{-1/2} \Phi (G_{K,x_1} ) - \Phi _{K}(x_1,t) \right) q_1^t U_K \Psi _{N,t} \big \rangle \end{aligned}$$

6.4b $$\begin{aligned}&\quad + 2\mathrm {Im}\big \langle U_K \Psi _{N,t} , p_1^t \Phi _{\geqq K}(x_1,t) q_1^t U_K \Psi _{N,t} \big \rangle \end{aligned}$$

6.4c $$\begin{aligned}&\quad - 2\mathrm {Im}\big \langle U_K \Psi _{N,t} , p_1^t A_{K,x_1} q_1^tU_K \Psi _{N,t} \big \rangle \end{aligned}$$

6.4d $$\begin{aligned}&\quad - 2\mathrm {Im}\big \langle U_K \Psi _{N,t} , p_1^t (N-1) V_K(x_1-x_2) q_1^t U_K \Psi _{N,t} \big \rangle . \end{aligned}$$

The various terms will be bounded as follows:

**Line** (6.4a). We bound

6.5 $$\begin{aligned} \left| (6.4\mathrm{a}) \right|&\leqq 2 \left| \big \langle \int _{\vert k \vert \leqq K } d^3k \, \vert k \vert ^{-1} e^{- ikx_1} \left( N^{-1/2} a^*_k - \overline{\varphi _t(k)} \right) p_1^t U_K \Psi _{N,t} , q_1^t U_K \Psi _{N,t} \big \rangle \right| \nonumber \\ {}&\quad + 2 \left| \big \langle \int _{\vert k \vert \leqq K } d^3k \, \left| k \right| ^{-1} e^{ikx_1} \left( N^{-1/2} a_k - \varphi _t(k) \right) p_1^t U_K\Psi _{N,t} , q_1^t U_K \Psi _{N,t} \big \rangle \right| \nonumber \\ {}&\leqq 2 \beta ^a_K (\Psi _{N,t},\psi _t) + \left\| \int _{\vert k \vert \leqq K } d^3k \, \left| k \right| ^{-1} e^{-ikx_1} \left( N^{-1/2} a_k^* - \overline{\varphi _t(k)} \right) p_1^t U_K \Psi _{N,t} \right\| ^2 \nonumber \\ {}&\quad + \left\| \int _{\vert k \vert \leqq K } d^3k \, \left| k \right| ^{-1} e^{i kx_1} \left( N^{-1/2} a_k - \varphi _t(k) \right) p_1^t U_K \Psi _{N,t} \right\| ^2. \end{aligned}$$

For the second summand, we use

6.6 $$\begin{aligned}&\left\| \int _{\vert k \vert \leqq K } d^3k \, \left| k \right| ^{-1} e^{-ikx_1} \left( N^{-1/2} a_k^* - \overline{\varphi _t(k)} \right) p_1^t U_K \Psi _{N,t} \right\| ^2 \nonumber \\ {}&\quad \leqq \frac{C K}{N} + \left\| \int _{\vert k \vert \leqq K } d^3 k \, \vert k \vert ^{-1} e^{ikx_1} \left( N^{-1/2} a_k - \varphi _t(k) \right) p_1^t U_K \Psi _{N,t} \right\| ^2 , \end{aligned}$$

which follows directly from the canonical commutation relations. By the shift property (3.10), the last summand in (6.5) can be written as

6.7 $$\begin{aligned} N^{-1} \left\| a \left( G_{K,x_1} \right) p_1^t W^*(\sqrt{N} \varphi _t) U_K \Psi _{N,t} \right\| ^2 . \end{aligned}$$

In order to estimate this expression, we use [10, Lemma 10] which implies the bound

6.8 $$\begin{aligned} a^*\left( G_{K,x_1} \right) a \left( G_{K,x_1} \right)&\leqq C_{G} \, \left( 1 - \Delta _1 \right) {\mathcal {N}} , \end{aligned}$$

with

6.9 $$\begin{aligned} C_G = \sup _{p\in {\mathbb {R}}^3} \int _{{\mathbb {R}}^3} \frac{d^3k}{k^2(1+(p+k)^2)}=\int _{{\mathbb {R}}^3} \frac{d^3k}{k^2(1+k)^2} <\infty . \end{aligned}$$

The latter is obtained via a rearrangement inequality. In combination, we thus have

6.10 $$\begin{aligned} \left| (6.4\mathrm{a}) \right|&\leqq C \left( \beta ^a_K (t) + K N^{-1} \right) + C_G \, N^{-1} \left\| \left( 1 - \Delta _1 \right) ^{1/2} p_1^t {\mathcal {N}}^{1/2} W^* ( \sqrt{N} \varphi _t ) U_K \Psi _{N,t} \right\| ^2 \nonumber \\&\leqq C \left( \beta ^a_K(t ) + K N^{-1} \right) + C_G \, \left\| \psi _t \right\| ^2_{H^1} N^{-1} \left\| {\mathcal {N}}^{1/2} W^* ( \sqrt{N} \varphi _t ) U_K \Psi _{N,t} \right\| ^2 \nonumber \\&\leqq C \left\| \psi _t \right\| ^2_{H^1} \left( \beta ^a_K(t ) + \beta ^b_K(t ) + K N^{-1} \right) . \end{aligned}$$

**Line** (6.4b). This term can be estimated as

6.11 $$\begin{aligned} \left| (6.4\mathrm{b}) \right|&\leqq C \sup _x \left| \Phi _{\ge K}(x,t) \right| \left\| q_1^t U_K \Psi _{N,t} \right\| \nonumber \\&\leqq C \sqrt{\beta ^a_K (t)} \int _{\left| k \right| \geqq K} d^3k \left| k \right| ^{-1} \left| \varphi _t \right| \nonumber \\&\leqq C \sqrt{\beta ^a_K (t)} \left\| \varphi _t \right\| _{L_1^2} \left( \int _{\left| k \right| \geqq K} d^3k \left| k \right| ^{-4} \right) ^{1/2} \leqq C \left\| \varphi _t \right\| _{L_1^2} \sqrt{\beta ^a_K (t)} K^{-1/2}. \end{aligned}$$

**Line** (6.4c). It follows from (5.20) that

6.12 $$\begin{aligned} \left| (6.4\mathrm{c}) \right| \leqq C \Big ( \left\| \psi _t \right\| _{H^1}^2 + \left\| \varphi _t \right\| _{L_1^2}^2 + \vert E_N^\mathrm {F}(\Psi _{N,t}) \vert \Big ) \left( K^{-1} + \beta _K^b(t) \right) . \end{aligned}$$

**Line** (6.4d). In analogy to (5.21) one finds

6.13 $$\begin{aligned} \left| (.6.4\mathrm{d}) \right| \leqq C \big ( \beta ^a_K(t ) + K^{-2}\big ) . \end{aligned}$$

In combination, we have thus shown that

6.14 $$\begin{aligned} \left| \frac{\mathrm {d}}{\mathrm {d}t}\beta ^a_K (t) \right| \leqq C \left( \left\| \psi _t \right\| _{H^1}^2 + \left\| \varphi _t \right\| _{L_1^2}^2 + \vert E^\mathrm {F}_N(\Psi _{N,t}) \vert \right) \left( \beta _K^a(t) + \beta ^b_K(t) + \frac{K}{N} + K^{-1} \right) . \end{aligned}$$

### Time Derivative of $\beta ^b_K(t)$

From (6.1) we get

6.15a $$\begin{aligned}&\frac{\mathrm{d}}{\mathrm{d}t} \beta _K^b(\Psi _{N,t},\varphi _t) \nonumber \\&\quad = \int d^3k \, \frac{\mathrm{d}}{\mathrm{d}t} \big \langle \left( N^{-1/2} a_k - \varphi _t(k)\right) U_K \Psi _{N,t} , \left( N^{-1/2} a_k - \varphi _t(k) \right) U_K \Psi _{N,t} \big \rangle \nonumber \\&\quad = - 2 \mathrm {Re}\int d^3k \, \big \langle \left( N^{-1/2} a_k^* - \overline{\varphi _t(k)} \right) \left( N^{-1/2} a_k - \varphi _t(k) \right) U_K \Psi _{N,t} , i H_{N,K}^\mathrm{G} U_K \Psi _{N,t} \big \rangle \end{aligned}$$

6.15b $$\begin{aligned}&\qquad - 2 \mathrm {Re}\int d^3k \, \big \langle \left( \partial _t \varphi _t(k) \right) U_K \Psi _{N,t} , \left( N^{-1/2} a_k - \varphi _t(k) \right) U_K \Psi _{N,t} \big \rangle ,\end{aligned}$$

which is a slightly formal computation, since the use of the product rule of differentiation is not completely obvious here. We clarify the difficulty and justify the above identity in detail in “Appendix B”. Next, we write the first line in terms of the commutator as

6.16 $$\begin{aligned}&(6.15\mathrm{a}) \nonumber \\ {}&\quad = - i \int d^3k \, \big \langle \big [ H_{N,K}^\mathrm {G} , \big ( N^{-1/2} a_k^* - \overline{\varphi _t(k)} \big ) \big ( N^{-1/2} a_k - \varphi _t(k) \big ) \big ] U_K \Psi _{N,t} , U_K \Psi _{N,t} \big \rangle . \end{aligned}$$

Let us remark that the right hand side is well defined since the commutator of $H_{N,K}^\mathrm{G}$ and

6.17 $$\begin{aligned} L_N = N^{-1} W \big ( \sqrt{N} \varphi _t \big ) {\mathcal {N}} W^* \big ( \sqrt{N} \varphi _t \big ) = \int d^3k \, \big ( N^{-1/2} a_k^* - \overline{\varphi _t(k)} \big ) \big ( N^{-1/2} a_k - \varphi _t(k) \big ) \end{aligned}$$

defines a bounded operator from ${\mathcal {D}} \big ( H_{N,K}^{ \mathrm G} \big ) = {\mathcal {D}} \big ( H_N^{0} \big )$ to ${\mathcal {H}}^{(N)}$. This fact is a direct consequence of the B.L.T. theorem because $\big [ H_{N,K}^\mathrm{G}, L_N \big ]$ is a bounded operator from ${\mathcal {D}} \big ( (H_N^0 )^2 \big )$ to ${\mathcal {H}}^{(N)}$ and the estimate

6.18 $$\begin{aligned} \left\| \big [ H_{N,K}^\mathrm{G}, L_N \big ] \Psi _N \right\| \leqq C_{N,K} \left\| \big (1 + H_{N}^0 \big ) \Psi _N \right\| \end{aligned}$$

holds for all $\Psi _N \in {\mathcal {D}} \big ( (H_N^0)^2 \big )$ and some constant $C_{N,K}$. The latter is straightforward to verify with the aid of

6.19 $$\begin{aligned} \left[ H_{N,K}^\mathrm{G} , a_k \right]&= 2 N^{-1/2} \sum _{j=1}^N B_{K,x_j}(k) k \cdot \left( i \nabla _j - N^{-1/2} \Phi \big ( k B_{K,x_j} \big ) \right) \nonumber \\&\quad - a_k - N^{-1/2} \sum _{j=1}^N \left| k \right| ^{-1} \mathbb {1}_{\left| k \right| \leqq K}(k) e^{-ikx_j} \end{aligned}$$

and the basic estimates from Section 3.

Hence, we can proceed by using (6.16) and (6.19) together with the Landau–Pekar equations (1.6) and the symmetry of the many-body wave function in order to obtain

6.20 $$\begin{aligned}&\frac{\mathrm{d}}{\mathrm{d}t} \beta ^b_K (\Psi _{N,t}, \varphi _t) \nonumber \\&\quad = - 2 \int _{\left| k \right| \leqq K} d^3k \, \left| k \right| ^{-1} \mathrm {Im}\big \langle e^{-ikx_1} U_K \Psi _{N,t} , \left( N^{-1/2} a_k - \varphi _t(k) \right) U_K \Psi _{N,t} \big \rangle \nonumber \\&\qquad + 2 \int d^3k \, \left| k \right| ^{-1} \mathrm {Im}\big \langle \int d^3y \, e^{-iky} \left| \psi _t(y) \right| ^2 U_K \Psi _{N,t} , \left( N^{-1/2} a_k - \varphi _t(k) \right) U_K \Psi _{N,t} \big \rangle \nonumber \\&\qquad + 4 \int d^3k \, \mathrm {Im}\big \langle B_{K,x_1}(k) k \cdot \left( i \nabla _1 - N^{-1/2} \Phi (k B_{K,x_1}) \right) U_K \Psi _{N,t} , \left( N^{-1/2} a_k - \varphi _t(k) \right) U_K \Psi _{N,t} \big \rangle . \end{aligned}$$

Finally, inserting the identity $e^{ikx_1} = p_1^t e^{ikx_1} p_1^t + q_1^t e^{ikx_1} p_1^t + e^{ikx_1} q_1^t$ leads to

6.21a $$\begin{aligned}&\frac{\mathrm {d}}{\mathrm {d}t} \beta ^b_K(\Psi _{N,t}, \varphi _t) \nonumber \\ {}&\quad = - 2 \int _{\left| k \right| \leqq K} d^3k \, \left| k \right| ^{-1} \mathrm {Im}\big \langle U_K \Psi _{N,t} , \Big ( p_1^t e^{ikx_1} p_1^t - \int d^3y \, e^{iky} \left| \psi _t(y) \right| ^2 \Big ) \nonumber \\ {}&\qquad \qquad \qquad \qquad \qquad \; \, \left( N^{-1/2} a_k - \varphi _t(k) \right) U_K \Psi _{N,t} \big \rangle \end{aligned}$$

6.21b $$\begin{aligned}&\qquad + 2 \int _{\left| k \right| \geqq K} d^3k \, \left| k \right| ^{-1} \mathrm {Im}\big \langle U_K \Psi _{N,t} , \int d^3y \, e^{iky} \left| \psi _t(y) \right| ^2 \left( N^{-1/2} a_k - \varphi _t(k) \right) U_K \Psi _{N,t} \big \rangle \end{aligned}$$

6.21c $$\begin{aligned}&\qquad - 2 \int _{\left| k \right| \leqq K} d^3k \, \left| k \right| ^{-1} \mathrm {Im}\big \langle U_K \Psi _{N,t} , q_1^t e^{ikx_1} p_1^t \left( N^{-1/2} a_k - \varphi _t(k) \right) U_K \Psi _{N,t} \big \rangle \end{aligned}$$

6.21d $$\begin{aligned}&\qquad - 2 \int _{\left| k \right| \leqq K} d^3k \, \left| k \right| ^{-1} \mathrm {Im}\big \langle U_K \Psi _{N,t} , e^{ikx_1} q_1^t \left( N^{-1/2} a_k - \varphi _t(k) \right) U_K \Psi _{N,t} \big \rangle \end{aligned}$$

6.21e $$\begin{aligned}&\qquad + 4 \int d^3k \, \mathrm {Im}\big \langle B_{K,x_1}(k) k \cdot \left( i \nabla _1 - N^{-1/2} \Phi \big ( k B_{K,x_1} \big ) \right) \nonumber \\ {}&\qquad \qquad \qquad \quad \; \; U_K \Psi _{N,t} , \left( N^{-1/2} a_k - \varphi _t(k) \right) U_K \Psi _{N,t} \big \rangle . \end{aligned}$$

In what follows, we estimate each term on the r.h.s. separately.

**Line** (6.21a). The first term is the most important because it is the one where the particle density cancels the source term of the Landau–Pekar equations. We first note that

6.22 $$\begin{aligned} p_1^t e^{ikx_1} p_1^t - \int d^3y \, e^{iky} \left| \psi _t(y) \right| ^2 = \left( p_1^t - 1 \right) \int d^3y \, e^{iky} \left| \psi _t(y) \right| ^2 = - q_1^t \big \langle \psi _t , e^{ ik \cdot } \psi _t \big \rangle . \end{aligned}$$

We then use $e^{ikx} = \frac{1 - i (k \cdot \nabla _x)}{1 + k^2} e^{ikx}$ and integrate by parts to obtain the bound

6.23 $$\begin{aligned} \left| \big \langle \psi _t , e^{ik \cdot } \psi _t \big \rangle \right|&\leqq \left| \big \langle \frac{1 - i ( k \cdot \nabla ) }{1 + k^2} \psi _t , e^{ik \, \cdot \,} \psi _t \big \rangle \right| + \left| \big \langle \psi _t , e^{ik \, \cdot \,} \frac{ i ( k \cdot \nabla )}{1 + k^2} \psi _t \big \rangle \right| \nonumber \\&\leqq 2 (1 + k^2)^{-1} \left( 1 + \left| k \right| \left\| \nabla \psi _t \right\| \right) \leqq 2 \left\| \psi _t \right\| _{H^1} (1 + k^2)^{-1} \left( 1 + \left| k \right| \right) . \end{aligned}$$

Hence,

6.24 $$\begin{aligned} \left| (6.21\mathrm{a}) \right|&\leqq 4 \left\| \psi _t \right\| _{H^1} \int d^3k \, \frac{(1+ \left| k \right| )}{\left| k \right| ( 1 + k^2)} \left| \big \langle q_1^t U_K \Psi _{N,t} , \left( N^{-1/2} a_k - \varphi _t(k) \right) U_K \Psi _{N,t} \big \rangle \right| \nonumber \\ {}&\leqq 4 \left\| \psi _t \right\| _{H^1} \sqrt{\beta ^a_K(t)} \Big ( \int d^3k \, \frac{(1+ \left| k \right| )^2}{\left| k \right| ^2 (1+ k^2)^2} \Big )^{1/2} \nonumber \\ {}&\quad \times \Big ( \int d^3k \, \left\| \left( N^{-1/2} a_k - \varphi _t(k) \right) U_K \Psi _{N,t} \right\| ^2 \Big )^{1/2} \nonumber \\ {}&\leqq C \left\| \psi _t \right\| _{H^1} \beta _K(t) . \end{aligned}$$

**Line** (6.21b). We again use (6.23) and estimate

6.25 $$\begin{aligned} (6.21\mathrm{b})&\leqq 2 \int _{\left| k \right| \geqq K} d^3k \, \left| k \right| ^{-1} \left| \big \langle \psi _t , e^{ik \cdot } \psi _t \big \rangle \right| \left| \big \langle U_K \Psi _{N,t} , \left( N^{-1/2} a_k - \varphi _t(k) \right) U_K \Psi _{N,t} \big \rangle \right| \nonumber \\&\leqq C \left\| \psi _t \right\| _{H^1} \int _{\left| k \right| \geqq K} d^3k \, \frac{(1+ \left| k \right| )}{\left| k \right| ( 1 + k^2)} \left\| \left( N^{-1/2} a_k - \varphi _t(k) \right) U_K \Psi _{N,t} \right\| \nonumber \\&\leqq C \left\| \psi _t \right\| _{H^1} \left( \beta _K^b(t) + \int _{\left| k \right| \geqq K} d^3k \, \frac{(1+ \left| k \right| )^2}{\left| k \right| ^2 ( 1 + k^2)^2} \right) \nonumber \\&\leqq C \left\| \psi _t \right\| _{H^1} \left( \beta _K^b(t) + K^{-1} \right) . \end{aligned}$$

**Line** (6.21c). Writing (6.21c) as

6.26 $$\begin{aligned} 2 \int _{\left| k \right| \leqq K} d^3k \, \left| k \right| ^{-1} \mathrm {Im}\big \langle e^{ikx_1} \left( N^{-1/2} a_k - \varphi _t(k) \right) p_1^t U_K \Psi _{N,t} , q_1^t U_K \Psi _{N,t} \big \rangle \end{aligned}$$

shows that this is exactly the same expression as the second line in (6.5). We consequently have

6.27 $$\begin{aligned} \left| (6.21\mathrm{c}) \right|&\leqq C \left\| \psi _t \right\| _{H^1}^2 \left( \beta ^a_K (t) + \beta ^b_K (t) \right) . \end{aligned}$$

**Line** (6.21d). To find a suitable bound for (6.21d) is the most difficult step in the proof. We start by estimating

6.28 $$\begin{aligned} \left| (6.21\mathrm{d}) \right|&\leqq 2 \int _{\left| k \right| \leqq K} d^3k \, \left| k \right| ^{-1} \Big | \big \langle U_K \Psi _{N,t} , e^{ikx_1} q_1^t \left( N^{-1/2} a_k - \varphi _t(k) \right) U_K \Psi _{N,t} \big \rangle \Big | \nonumber \\&= 2 \int _{\left| k \right| \leqq K} d^3k \, \left| k \right| ^{-1} \Big | \big \langle U_K \Psi _{N,t} , N^{-1} \sum _{j=1}^N e^{ikx_j} q_j^t \left( N^{-1/2} a_k - \varphi _t(k) \right) U_K \Psi _{N,t} \big \rangle \Big | \nonumber \\&\leqq 2 \int _{\left| k \right| \leqq K} d^3k \, \left| k \right| ^{-1} \Big \Vert N^{-1} \sum _{j=1}^N q_j^t e^{-ikx_j} U_K \Psi _{N,t} \Big \Vert \left\| \left( N^{-1/2} a_k - \varphi _t(k) \right) U_K \Psi _{N,t} \right\| \nonumber \\&\leqq \beta ^b_K(t) + \int _{\left| k \right| \leqq K} d^3k \, \left| k \right| ^{-2} \Big \Vert N^{-1} \sum _{j=1}^N q_j^t e^{-ikx_j} U_K \Psi _{N,t} \Big \Vert ^2 . \end{aligned}$$

The last term is bounded by

6.29 $$\begin{aligned}&\int _{\left| k \right| \leqq K} d^3k \, \left| k \right| ^{-2} \Big \Vert N^{-1} \sum _{j=1}^N q_j^t e^{-ikx_j} U_K \Psi _{N,t} \Big \Vert ^2 \nonumber \\ {}&\quad \leqq 4 \pi N^{-1} K + \int _{\left| k \right| \leqq K} d^3k \, \left| k \right| ^{-2} \left| \mathrm {Re}\, \big \langle q_2^t e^{-ikx_2} U_K \Psi _{N,t} , q_1^t e^{- ik x_1} U_K \Psi _{N,t} \big \rangle \right| . \end{aligned}$$

With $O_{1,2} = \left( 1- \Delta _2 \right) ^{-1/2} e^{ikx_2} \left( 1 - \Delta _1 \right) ^{1/2} q_2^t$ one has

6.30 $$\begin{aligned} e^{ ikx_2} q_2^t q_1^t e^{- ikx_1} + e^{ ikx_1} q_1^t q_2^t e^{- ikx_2}&= O_{2,1}^* O_{1,2} + O_{1,2}^* O_{2,1} \nonumber \\&\leqq O_{1,2}^* O_{1,2} + O_{2,1}^* O_{2,1}. \end{aligned}$$

Thus, using the symmetry of the wave function and $e^{-ikx_2} ( 1 - \Delta _2 )^{-1} e^{ikx_2} = ( ( - i \nabla _2 + k )^2 + 1 )^{-1}$, we obtain the bound

6.31 $$\begin{aligned}&\int _{\left| k \right| \leqq K} d^3k \, \left| k \right| ^{-2} \left| \mathrm {Re}\, \big \langle q_2^t e^{-ikx_2} U_K \Psi _{N,t} , q_1 e^{- ik x_1} U_K \Psi _{N,t} \big \rangle \right| \nonumber \\ {}&\quad \leqq \int _{\left| k \right| \leqq K} d^3k \, \left| k \right| ^{-2} \big \langle U_K \Psi _{N,t} , q_2^t \left( 1 - \Delta _1 \right) ^{1/2} \nonumber \\ {}&\qquad \qquad \qquad \qquad \; \, e^{- ikx_2} \left( 1 - \Delta _2 \right) ^{-1} e^{ ikx_2} \left( 1 - \Delta _1 \right) ^{1/2} q_2^t U_K \Psi _{N,t} \big \rangle \nonumber \\ {}&\quad = \big \langle \left( 1 - \Delta _1 \right) ^{1/2} q_2^t U_K \Psi _{N,t} , \int _{\left| k \right| \leqq K} d^3k \, \nonumber \\ {}&\qquad \; \; \left| k \right| ^{-2} \left( \left( - i \nabla _2 + k \right) ^2 + 1 \right) ^{-1} \left( 1 - \Delta _1 \right) ^{1/2} q_2^t U_K \Psi _{N,t} \big \rangle . \end{aligned}$$

In combination with

6.32 $$\begin{aligned}&\left\| \int _{\left| k \right| \leqq K} d^3k \, \left| k \right| ^{-2} \left( \left( - i \nabla _2 + k \right) ^2 + 1 \right) ^{-1} \right\| _{\mathrm {op}} \nonumber \\&\quad = \sup _{p\in {\mathbb {R}}^3} \int _{\left| k \right| \leqq K} d^3k \, \left| k \right| ^{-2} \left( \left( p + k \right) ^2 + 1 \right) ^{-1} < \infty \end{aligned}$$

(compare with (6.9)) and Lemma 5.1 this gives

6.33 $$\begin{aligned} \left| (6.21\mathrm{d}) \right|&\leqq C \left( \beta ^b_K(t) + N^{-1} K + \left\| (1 - \Delta _2)^{1/2} q_1^t U_K \Psi _{N,t} \right\| ^2 \right) \nonumber \\&\leqq C \left( \beta ^a_K (t) + \beta ^b_K (t) + N^{-1} K + \left\| \nabla _2 q_1^t U_K \Psi _{N,t} \right\| ^2 \right) \nonumber \\&\leqq C \left( \left\| \psi _t \right\| _{H^2}^2 + \left\| \varphi _t \right\| _{L_1^2}^2 + \vert E_N^\mathrm{{F}}(\Psi _{N,t}) \vert \right) \left( \beta _K(t) + N^{-1} K^{-1} + N^{-1} K \right) . \end{aligned}$$

**Line** (6.21e). We have

6.34 $$\begin{aligned} \left| (6.21\mathrm{e}) \right|&\leqq 4 \int d^3k \, \left| k \right| \left| B_{K,x}(k) \right| \left\| \left( N^{-1/2} a_k - \varphi _t(k) \right) U_K \Psi _{N,t} \right\| \nonumber \\&\qquad \times \left\| \left( i \nabla _1 - N^{-1/2} \Phi \big ( k B_{K,x_1} \big ) \right) U_K \Psi _{N,t} \right\| \nonumber \\&\leqq C \sqrt{\beta ^b_K(t)} \left\| \left( i \nabla _1 - N^{-1/2} \Phi \big ( k B_{K,x_1} \big ) \right) U_K \Psi _{N,t} \right\| \Vert \left| \cdot \right| B_{K,x} \Vert . \end{aligned}$$

By using (3.7), the symmetry of the wave function and (3.20), we get

6.35 $$\begin{aligned} \left| (6.21\mathrm{e}) \right|&\leqq C \left( \left\| \nabla _1 U_K \Psi _{N,t} \right\| + \left\| \left| \cdot \right| B_{K,x_1} \right\| \left\| N^{-1/2} \left( {\mathcal {N}} +1 \right) U_K \Psi _{N,t} \right\| \right) \left( \beta ^b_K(t) + K^{-1} \right) \nonumber \\&\leqq C \left( \big \langle U_K \Psi _{N,t} , N^{-1} H_N^0 U_K \Psi _{N,t} \big \rangle ^{1/2} + 1 \right) \left( \beta ^b_K(t) + K^{-1} \right) \nonumber \\&\leqq C \left( \big \langle U_K \Psi _{N,t} , ( N^{-1} H_{N,K}^\mathrm{G} +C) U_K \Psi _{N,t} \big \rangle ^{1/2} + 1 \right) \left( \beta ^b_K(t) + K^{-1} \right) . \end{aligned}$$

In total, we thus arrive at

6.36 $$\begin{aligned} \left| \frac{\mathrm{d}}{\mathrm{d}t} \beta ^b_K(\Psi _{N,t}, \varphi _t) \right|&\leqq C \left( \left\| \psi _t \right\| _{H^2}^2 + \left\| \varphi _t \right\| _{L_1^2}^2 + \vert E_N^\mathrm{{F}}(\Psi _{N,t}) \vert \right) \left( \beta _K(t) + K^{-1} + \frac{K}{N} \right) . \end{aligned}$$

**Conclusion:** We combine $\frac{\mathrm{d}}{\mathrm{d}t}\beta ^c(t) = 0$, (6.14) and (6.36) with Proposition (2.1) and $\vert E_N^\mathrm{F}(\Psi _{N,t}) \vert = \vert E_N^\mathrm{F}(\Psi _N) \vert \leqq E_0$ in order to obtain (4.15).$\square$

## Remaining Proofs

### Proof of Lemma 4.1

To show the inequality (4.1), we use $\gamma ^{(1,0)}_{U_K\Psi _N} = \gamma ^{(1,0)}_{U_{K,x_1}\Psi _N}$ with $U_{K,x_1} = \exp (i N^{-1/2} \Pi (B_{K ,x_1}))$, which follows directly from (3.11) and the definition of the reduced density matrix. Hence,

7.1 $$\begin{aligned} \text{ Tr}_{L^2({\mathbb {R}}^3)} \Big \vert \gamma ^{(1,0)}_{\Psi _N} - \gamma ^{(1,0)}_{U_K\Psi _N} \Big \vert \leqq 2 \left\| (U_{K,x_1}-1) \Psi _{N} \right\| . \end{aligned}$$

Using $\left\| (U_{K,x_1}-1) \Psi _{N} \right\| = \left\| (U_{K,x_1}-1) U_K \Psi _{N} \right\|$, we obtain (4.1) from the bound

7.2 $$\begin{aligned} \left\| ( U_{K,x_1} -1) U_K \Psi _N \right\| \leqq 2 \left\| B_{K,x} \right\| \, \left\| \Big (\frac{{\mathcal {N}}+1}{N} \Big )^{1/2} U_K \Psi _N \right\| , \end{aligned}$$

together with ${\mathcal {N}} \leqq H_N^0$, (3.20) and (3.18). Inequality (7.2) follows from the spectral calculus for self-adjoint operators, using $1-U_{K,x}=f(N^{-1/2}\Pi (B_{K,x}))$ with $f(s) = 1-\exp (i s)$ in combination with $\vert f(s) \vert \leqq \vert s \vert$.

Using the properties of the Weyl operator (in particular (3.9) together with (3.11)) and

7.3 $$\begin{aligned} U_K{\mathcal {N}} U^*_K&= {\mathcal {N}} + N^{-1} \sum _{i,j=1}^N \big \langle B_{K,x_i} , B_{K,x_j} \big \rangle + N^{-1/2} \sum _{j=1}^N \left( a ( B_{K, x_j} ) + a^* ( B_{K, x_j} ) \right) , \end{aligned}$$

we have

7.4 $$\begin{aligned}&N^{-1} \left| \big \langle W^*(\sqrt{N} \varphi ) \Psi _{N} , \big ( {\mathcal {N}} - U_K^* {\mathcal {N}} U_K \big ) W^* (\sqrt{N} \varphi ) \Psi _N \big \rangle \right| \nonumber \\&\quad = N^{-1} \Big | \big \langle W^*(\sqrt{N} \varphi ) U_K \Psi _{N} , \big ( U_K {\mathcal {N}} U^*_K -{\mathcal {N}} \big ) W^*(\sqrt{N} \varphi ) U_K \Psi _N \big \rangle \nonumber \\&\quad \leqq \left\| B_{K,x} \right\| ^2 + 2 N^{-3/2} \sum _{j=1}^N \left\| a\left( B_{K,x_j} \right) W^*(\sqrt{N} \varphi ) U_K \Psi _N \right\| \nonumber \\&\quad \leqq \left\| B_{K,x} \right\| ^2 + 2 \left\| B_{K,x} \right\| \left\| \varphi \right\| + 2 N^{-1/2} \left\| B_{K,x} \right\| \left\| {\mathcal {N}}^{1/2} U_K \Psi _N \right\| . \end{aligned}$$

An application of (3.7) and (3.20) then leads to

7.5 $$\begin{aligned} (7.4)&\leqq C (1+\left\| \varphi \right\| ) \left\| B_{K,x} \right\| \big ( 1 + \big \langle U_K \Psi _N , N^{-1} H_N^0 U_K \Psi _N \big \rangle ^{1/2} \big ) \nonumber \\&\leqq C K^{-3/2} ( 1+\left\| \varphi \right\| ) \big \langle U_K \Psi _N , ( N^{-1} H_{N,K}^\mathrm{G} + C ) U_K \Psi _N \big \rangle ^{1/2}. \end{aligned}$$

In combination with (3.18), this shows (4.2). $\square$

### Proof of Proposition 2.2

Throughout this section, we set $\xi _N = \psi ^{\otimes N}\otimes W(\sqrt{N} \varphi ) \Omega$. The bound on the energy follows from

7.6 $$\begin{aligned} \big \langle \Psi _{N} , H_N^\mathrm{F} \Psi _N \big \rangle = \big \langle \xi _{N} , H_{N,K}^\mathrm{G} \xi _{N} \big \rangle = \big \langle \xi _{N} , \Big ( H_{N,K}^\mathrm{F} + \sum _{j=1}^N A_{K,x_j} + \sum _{j,l=1}^N V_{K}(x_j-x_l) \Big ) \xi _{N} \big \rangle \end{aligned}$$

in combination with (3.17), (A.2) and

7.7 $$\begin{aligned} \left| \big \langle \xi _{N} , H_{N,K}^\mathrm{F} \xi _{N} \big \rangle \right|&= N \left| \big \langle \psi , \left( - \Delta + \Phi _{K}(\cdot ,0) \right) \psi \big \rangle + \left\| \varphi \right\| ^2 \right| \leqq C N \left( \left\| \psi \right\| _{H^1}^2 + \left\| \varphi \right\| ^2 \right) . \end{aligned}$$

In (7.7), we used the shift property of the Weyl operators (3.10).

For the bound on $a(\Psi _{N},\psi )$, we note that

7.8 $$\begin{aligned} \text {Tr}_{L^2({\mathbb {R}}^3)} \left| \gamma ^{(1,0)}_{\Psi _{N}} - | \psi \rangle \langle \psi | \right| = \text {Tr}_{L^2({\mathbb {R}}^3)} \left| \gamma ^{(1,0)}_{\Psi _{N}} - \gamma ^{(1,0)}_{ U_K \Psi _{N}} \right| \end{aligned}$$

since $| \psi \rangle \langle \psi | = \gamma ^{(1,0)}_{\xi _{N}}$ and $\xi _{N} = U_K\Psi _{N}$. Applying Lemma (4.1), we obtain the stated estimate. The bound on $b(\Psi _{N},\varphi )$ follows readily from (3.12),

7.9 $$\begin{aligned} b(\Psi _{N},\varphi )&= N^{-1} \int d^3k \, \left\| a_k \, U^{*}_K ( \psi ^{\otimes N} \otimes \Omega ) \right\| ^2 \leqq \left\| B_{K,x} \right\| ^2 \leqq C K^{-3} . \end{aligned}$$

We are thus left with the bound for $c(\Psi _{N})$, which we write with the aid of (3.18) as

7.10 $$\begin{aligned} c(\Psi _{N}) = \left\| N^{-1} \left( H_{N,K}^\mathrm{G} - \big \langle \xi _{N} , H_{N,K}^\mathrm{G} \xi _{N} \big \rangle \right) \xi _{N} \right\| ^2. \end{aligned}$$

Recalling Definition 3.1 and using the triangle inequality, we get

7.11 $$\begin{aligned} \left\| \left( H_{N,K}^\mathrm {G} - \big \langle \xi _{N} , H_{N,K}^\mathrm {G} \xi _{N} \big \rangle \right) \xi _{N} \right\|&\leqq \left\| \left( H_{N,K}^\mathrm {F} - \big \langle \xi _{N} , H_{N,K}^\mathrm {F} \xi _{N} \big \rangle \right) \xi _{N} \right\| \nonumber \\ {}&+ 2 \left\| \sum _{j=1}^N A_{K,x_j} \xi _{N} \right\| + 2 \left\| \sum _{j,l=1}^NV_K(x_j-x_l) \xi _N \right\| .\end{aligned}$$

After a lengthy but straightforward computation, using the shift property (3.10) and the fact that $\Delta _x$ commutes with $W(\sqrt{N} \varphi )$, we find that

7.12 $$\begin{aligned}&\frac{1}{N} \left\| \left( H_{N,K}^\mathrm{F} - \big \langle \xi _{N} , H_{N,K}^\mathrm{F} \xi _{N} \big \rangle \right) \xi _{N} \right\| ^2 \nonumber \\&\quad = \left\| \varphi \right\| ^2 + \big \langle \psi , (-\Delta )^2\psi \big \rangle - \big \langle \psi , (-\Delta )\psi \big \rangle ^2 + N^{-1} \big \langle \psi , \left\| G_{K,x} \right\| ^2\psi \big \rangle \nonumber \\&\quad +(1-N^{-1}) \left\| \big \langle \psi , G_{K,x}\psi \big \rangle \right\| ^2 + 4 \big \langle \psi , (\mathrm {Re}\big \langle G_{K,x} , \varphi \big \rangle )^2\psi \big \rangle - 4 \big \langle \psi , \mathrm {Re}\big \langle G_{K,x} , \varphi \big \rangle \psi \big \rangle ^2 \nonumber \\&\quad +2 \mathrm {Re}\big \langle \psi , \big \langle \varphi , G_{K,x} \big \rangle \psi \big \rangle + 2 \big ( \big \langle \psi , (-\Delta _x)\mathrm {Re}\big \langle G_{K,x} , \varphi \big \rangle \psi \big \rangle + \text {c.c.} \big ) \nonumber \\&\quad - 4 \big \langle \psi , (-\Delta )\psi \big \rangle \big \langle \psi , \mathrm {Re}\big \langle G_{K,x} , \varphi \big \rangle \psi \big \rangle . \end{aligned}$$

We shall show that the right hand side is bounded from above by a constant times $1+K N^{-1}$, with the constant depending only on $\left\| \psi \right\| _{H^2}$ and $\left\| \varphi \right\| _{L^2_1}$. For the first four summands (i.e., the terms in the first line), this is obvious (recall (3.7)). In the fifth summand, we can use (6.23) to conclude that

$\left\| \big \langle \psi , G_{K,x}\psi \big \rangle \right\| ^2\leqq C$ independently of *K*. For each of the remaining terms on the right side of (7.12), we use

7.13 $$\begin{aligned} \left| \big \langle G_{K,x} , \varphi \big \rangle \right| \leqq \left\| (1+ \left| \,\cdot \, \right| )^{-1} G_{K,x} \right\| _2 \, \left\| (1+ \left| \,\cdot \, \right| ) \varphi \right\| , \end{aligned}$$

which is bounded by $C \left\| \varphi \right\| _{L_1^2}$. Hence, we find

7.14 $$\begin{aligned} \left\| N^{-1} \left( H_{N,K}^\mathrm {F} - \big \langle \xi _{N} , H_{N,K}^\mathrm {F} \xi _{N} \big \rangle \right) \xi _{N} \right\| ^2 \leqq C\big ( N^{-1} + KN^{-2} \big ). \end{aligned}$$

Next, we use

7.15 $$\begin{aligned} \frac{1}{N} \left\| \sum _{j=1}^N A_{K,x_j} \xi _N \right\| \leqq \left\| A_{K,x_1}\xi _N \right\| , \end{aligned}$$

and recalling (3.13) we estimate (with ${\widetilde{\Psi }}_N = \psi ^{\otimes N}\otimes \Omega$)

7.16 $$\begin{aligned}&\left\| N^{-1/2}\nabla _1 \cdot a \big ( k B_{K,x_1} \big ) W (\sqrt{N} \varphi ) {\widetilde{\Psi }}_N \right\| \nonumber \\ {}&\quad = \left\| \nabla _1 \cdot \int dk\, k \overline{B_{K,x_1}(k) } \varphi (k) {\widetilde{\Psi }}_N \right\| \nonumber \\ {}&\quad \leqq \left\| \int dk \, k^2 \overline{B_{K,x_1}(k) } \varphi (k) {\widetilde{\Psi }}_N \right\| + \left\| \int dk\, k \overline{B_{K,x_1}(k) } \varphi (k) \nabla _1 {\widetilde{\Psi }}_N \right\| \nonumber \\ {}&\quad \leqq \left\| \left| \cdot \right| B_{K, x } \right\| _2 \big ( \left\| \left| \cdot \right| \varphi \right\| + \left\| \varphi \right\| \left\| \psi \right\| _{H^1} \big ) \leqq CK^{-1/2}. \end{aligned}$$

Similarly, we also have

7.17 $$\begin{aligned} \left\| N^{-1/2} a^* \big ( k B_{K,x_1} \big ) \cdot \nabla _1 W (\sqrt{N} \varphi ) {\widetilde{\Psi }}_N \right\|&= \left\| \int dk \,k \, B_{K_N,x_1}(k) \big ( N^{-1/2}a_k^* + \overline{\varphi (k)}\big ) \cdot \nabla _1 {\widetilde{\Psi }}_N \right\| \nonumber \\ {}&\leqq \left\| \left| \cdot \right| B_{K,x} \right\| \big ( N^{-1/2} \left\| \psi \right\| _{H^1} + \left\| \varphi \right\| \left\| \nabla \psi \right\| \big ) \leqq C K^{-1/2} . \end{aligned}$$

In order to estimate the term containing $\Phi (kB_{K,x})^2$, consider

7.18 $$\begin{aligned} W^*(\sqrt{N} \varphi ) \Phi (k B_{K ,x_1}) W(\sqrt{N} \varphi )&= \Phi (k B_{K ,x_1}) + 2 \sqrt{N}\mathrm {Re}\big \langle kB_{K ,x_1} , \varphi \big \rangle , \end{aligned}$$

and thus

7.19 $$\begin{aligned}&\left\| N^{-1 }\Phi (k B_{K,x_1})^2 W(\sqrt{N}\varphi ) {\widetilde{\Psi }}_N \right\| \nonumber \\ {}&\quad = N^{-1} \left\| \Big ( \Phi (k B_{K ,x_1}) + 2 \sqrt{N}\mathrm {Re}\big \langle k B_{K ,x_1} , \varphi \big \rangle \Big )^2 {\widetilde{\Psi }}_N \right\| \nonumber \\ {}&\quad \leqq 2 N^{-1 }\left\| \Phi (k B_{K ,x_1})^2 {\widetilde{\Psi }}_N \right\| + 8 \vert \big \langle {|} k B_{K ,x_1} {|} , {|} \varphi {|} \big \rangle \vert ^2. \end{aligned}$$

In the last line, we use (3.7) to obtain

7.20 $$\begin{aligned} \left\| \Phi (k B_{K, {x_1}})^2 {\widetilde{\Psi }}_N \right\| = \sqrt{3} \left\| k B_{K , {x}} \right\| ^2 \leqq C K^{-1}. \end{aligned}$$

Finally, using (3.17), we estimate

7.21 $$\begin{aligned} N^{-1} \left\| \sum _{j,l=1}^N V_K(x_j-x_l) \xi _N \right\| \leqq N^{-1} \sum _{j,l=1}^N\left\| V_K(x_j-x_l) \xi _N \right\| \leqq C K^{-1}, \end{aligned}$$

which completes the proof of the proposition. $\square$

### Proof of Theorem 2.2

Given Theorem 2.1, (2.19) follows from (4.1) together with the bound

7.22 $$\begin{aligned} \left\| (1-U_K) \Psi _{N} \right\| \leqq \frac{C N}{ K^{3/2} } \left\| \Big ( \frac{H_{N,K}^\mathrm {G} + C N }{N}\Big )^{1/2} \Psi _{N} \right\| ,\quad \Psi _N \in {\mathcal {D}} ( H_N^0 ). \end{aligned}$$

The latter follows from ${\mathcal {N}} \leqq H^0_N$, (3.20) and the functional calculus for self-adjoint operators, using $1-U_K = f( N^{-1/2} \sum _{j=1}^N \Pi (B_{K,x_j} ) )$ with $f(s) = 1-\exp (is)$ and the bound $\vert f(s) \vert \leqq \vert s \vert$. In more detail, let $\Psi _{N,t}$ as in Theorem 2.2 and denote $\Phi _{N,t} = e^{- i H_N^\mathrm{F} t} U^*_K \Psi _{N,0}$. Then, using (7.22),

7.23 $$\begin{aligned} \text{ Tr}_{L^2({\mathbb {R}}^3)} \left| \gamma ^{(1,0)}_{\Psi _{N,t}} - \gamma ^{(1,0)}_{\Phi _{N,t} } \right|&\leqq 2 \left\| e^{-iH_N^\mathrm {F} t } (\Psi _{N,0} - \Phi _{N,0} ) \right\| = 2 \left\| (1-U_K) \Psi _{N,0} \right\| \nonumber \\&\leqq \frac{CN}{K^{3/2}}, \end{aligned}$$

and the triangle inequality,

7.24 $$\begin{aligned} \text{ Tr}_{L^2({\mathbb {R}}^3)} \left| \gamma ^{(1,0)}_{\Psi _{N,t}} - | \psi _t \rangle \langle \psi _t | \right| \leqq \frac{C N}{K^{3/2}} + \text{ Tr}_{L^2({\mathbb {R}}^3)} \left| \gamma ^{(1,0)}_{\Phi _{N,t} } - | \psi _t \rangle \langle \psi _t | \right| . \end{aligned}$$

Since $\Phi _{N,0} \in {\mathcal {D}} ( H_N^\mathrm{F} )$ and $E_0 = \sup _{N \in {\mathbb {N}}} \vert N^{-1} \big \langle \Phi _{N,0} , H_N^\mathrm{F} \Phi _{N,0} \big \rangle \vert < \infty$ by assumption, we infer with Theorem 2.1 that

7.25 $$\begin{aligned}&\text{ Tr}_{L^2({\mathbb {R}}^3)} \left| \gamma ^{(1,0)}_{\Phi _{N,t}} - | \psi _t \rangle \langle \psi _t | \right| \nonumber \\ {}&\quad \leqq \sqrt{a (\Phi _{N,0 },\psi ) + b (\Phi _{N,0},\varphi ) + c (\Phi _{N,0}) + N^{-1/2} } e^{C(1 + \left| t \right| )^3}. \end{aligned}$$

Using Lemma 4.1, we have

7.26 $$\begin{aligned} a(\Phi _{N,0},\psi ) \leqq a(\Psi _{N,0 },\psi ) + C K^{-3/2} , \quad b(\Phi _{N,0 },\varphi ) \leqq b(\Psi _{N,0} ,\varphi ) + CK^{-3/2}, \end{aligned}$$

which proves the first bound in Theorem 2.2 if we set $K=K_N\ge c N^{5/6}$.

In order to prove (2.20), we estimate

7.27a $$\begin{aligned}&\big \langle W^* (\sqrt{N} \varphi _t) \Psi _{N,t} , \sqrt{ \tfrac{{\mathcal {N}}}{N} } \, W^* (\sqrt{N} \varphi _t) \Psi _{N,t} \big \rangle \nonumber \\ {}&\quad \leqq \big \vert \big \langle W^{*} (\sqrt{N} \varphi _t) \Psi _{N,t} , \sqrt{ \tfrac{{\mathcal {N}}}{N} } \, W^{*} (\sqrt{N} \varphi _t) \Phi _{N,t} \big \rangle \big \vert \end{aligned}$$

7.27b $$\begin{aligned}&\quad + \big \vert \big \langle W^{*} (\sqrt{N} \varphi _t) \Psi _{N,t} , \sqrt{ \tfrac{{\mathcal {N}}}{N} } \, W^{*} (\sqrt{N} \varphi _t) (\Psi _{N,t}-\Phi _{N,t} ) \big \rangle \big \vert \end{aligned}$$

with $\Phi _{N,t}$ defined as above. In the first line, we use the Cauchy–Schwarz inequality and apply Theorem 2.1 to $\Phi _{N,t}$, i.e.,

7.28 $$\begin{aligned} \left\| \sqrt{ \frac{{\mathcal {N}}}{N} } W^*(\sqrt{N} \varphi _t) \Phi _{N,t} \right\| ^2&\leqq \Big ( a (\Phi _{N,0},\psi ) + b (\Phi _{N,0},\varphi ) + c(\Phi _{N,0}) + N^{-1/2} \Big ) e^{C(1 + \left| t \right| )^3} \nonumber \\ {}&\leqq \Big ( a (\Psi _{N,0},\psi ) + b (\Psi _{N,0},\varphi ) + c(\Phi _{N,0}) + K^{-3/2} + N^{-1/2} \Big ) e^{C(1 + \left| t \right| )^3}, \end{aligned}$$

where we made use of (7.26) in the second step. In (7.27b), we estimate

7.29 $$\begin{aligned} \left\| \Psi _{N,t} - \Phi _{N,t} \right\| \leqq \left\| (1-U_{K}) \Psi _{N,0} \right\| \leqq \frac{C N}{ K^{3/2} } \left\| \Big ( \frac{H_{N,K}^\mathrm {G} +C N }{N}\Big )^{1/2} \Psi _{N,0} \right\| ,\end{aligned}$$

together with

7.30 $$\begin{aligned} \left\| \sqrt{ \frac{{\mathcal {N}}}{N} } W^*(\sqrt{N} \varphi ) \Psi _{N,t} \right\| \leqq C\Bigg ( \left\| \Big ( \frac{H_{N,K}^\mathrm {G} +C N}{N} \Big )^{1/2} \Psi _{N,0} \right\| + \left\| \varphi _t \right\| \Bigg ), \end{aligned}$$

which for $K=K_N\geqq c N^{5/6}$ proves (2.20). In order to show (7.30), we use the commutation relations (3.10) and $2 \Phi (\sqrt{N} \varphi _t) \leqq {\mathcal {N}} + N\left\| \varphi _t \right\| ^2$, in order to find

7.31 $$\begin{aligned} \big \langle W^*(\sqrt{N} \varphi _t) \Psi _{N,t} , {\mathcal {N}} W^*(\sqrt{N} \varphi _t) \Psi _{N,t} \big \rangle \leqq 2 \big ( \big \langle \Psi _{N,t} , {\mathcal {N}} \Psi _{N,t} \big \rangle + N \left\| \varphi _t \right\| ^2 \big ).\end{aligned}$$

Using (3.19) in combination with (3.20) leads to

7.32 $$\begin{aligned} {\mathcal {N}} \leqq H_N^0 \leqq 2 H_N^\mathrm {F} + C N&= e^{- iH_N^{ \mathrm F} t} (2 H_N^\mathrm {F} + C N) e^{i H_N^\mathrm {F} t } \nonumber \\&\leqq C e^{- iH_N^{ \mathrm F} t} ( H_{N,K}^\mathrm {G} + C N) e^{i H_N^\mathrm {F} t }. \end{aligned}$$

It remains to show (2.21) and (2.22): For the Pekar state $\Psi _{N} = \psi ^{\otimes N} \otimes W(\sqrt{N} \varphi ) \Omega$, we have

7.33 $$\begin{aligned} a(\Psi _{N},\psi ) = 0, \quad b(\Psi _{N},\varphi ) =0, \quad c( U_{K}^* \Psi _{N} ) \leqq C \Big (N^{-1} + K^{-1} + \frac{K}{N^2} \Big ), \end{aligned}$$

where the last bound was proven in Proposition 2.2. Thus, if we choose $K= K_N = c N$, we obtain (2.21) and (2.22). $\square$

## Auxiliary Bounds

In this appendix, we collect bounds on the interaction terms of the Hamiltonians $H_N^\mathrm{F}$ and $H_{N,K}^\mathrm{G}$ and derive the frequently used inequalities (3.19) and (3.20). After that we comment on the proof of Lemma 3.1.

### Lemma A.1

For every $\varepsilon >0$, $K \in (0,\infty ]$, $N \in {\mathbb {N}}$ and $j \in \{1,\ldots ,N\}$, we have

A.1 $$\begin{aligned} \pm N^{-1/2} \Phi (G_{K,x_j})&\leqq \varepsilon \left( - \Delta _j + \frac{ {\mathcal {N}}+1}{N} \right) +2 \frac{(16\pi )^2}{\varepsilon ^3} \end{aligned}$$

on $L^2({\mathbb {R}}^{3N}) \otimes {\mathcal {F}}_s$. Moreover, with $A_{K,x}$ defined in Definition 3.1,

A.2 $$\begin{aligned} \pm A_{K,x_j}&\leqq \sqrt{\frac{64 \pi }{K}} \left( - \Delta _j + N^{-1} {\mathcal {N}} \right) + \frac{16\pi }{N K} . \end{aligned}$$

### Proof

To prove (A.1), we use again the commutator method by Lieb and Yamazaki [26]. Using (3.4) and (3.7), we have

A.3 $$\begin{aligned} \left| \big \langle \Psi _N , N^{-1/2}\Phi (G_{K,x_j}) \Psi _N \big \rangle \right|&\leqq \frac{4\pi K }{\varepsilon } \left\| \Psi _N \right\| ^2 + {\varepsilon }N^{-1} \big \langle \Psi _N , ({\mathcal {N}}+1) \Psi _N \big \rangle , \end{aligned}$$

which proves (A.1) for $K\leqq 64 \pi /\varepsilon ^2$. In the case $K>64 \pi /\varepsilon ^2$, we write $\Phi (G_{K,x_j}) = \Phi (G_{K',x_j}) + ( \Phi (G_{K,x_j}) - \Phi (G_{K',x_j}) )$ with $K' = 16 \pi /\varepsilon ^2$. For the first summand, we use (A.3) with *K* replaced by $K'$ and $\varepsilon$ replaced by $\varepsilon /2$, while for the remainder, write

A.4 $$\begin{aligned} \big \langle \Psi _N , ( \Phi (G_{K,x_j}) -\Phi (G_{K',x_j}) )\Psi _N \big \rangle = \big \langle \Psi _N , [\nabla _j, \Phi (g_{x_j}) ] \Psi _N \big \rangle \end{aligned}$$

with $g_x(k) = i k \vert k \vert ^{-3}e^{-ikx} \mathbb {1}_{K'\leqq \vert k \vert \leqq K}(k)$. The absolute value of the last expression is bounded from above by

A.5 $$\begin{aligned} 4 \left\| \nabla _j \Psi _N \right\| \Vert g_x\Vert \left\| \sqrt{ {\mathcal {N}}+1} \Psi _N \right\| \leqq 2 N^{1/2} \left\| g_x \right\| \big \langle \Psi _N , \Big (-\Delta _j + \frac{{\mathcal {N}} + 1}{N} \Big ) \Psi _N \big \rangle . \end{aligned}$$

Using $$\left\| g_x \right\| \le \sqrt{4\pi / K'} = \frac{\varepsilon }{4}$$ shows (A.1).

To show (A.2), we use $\left\| \left| \cdot \right| B_{K,x_j} \right\| ^2 \leqq 4 \pi K^{-1}$ and

A.6 $$\begin{aligned}&\left| \big \langle \Psi _N , A_{K,x_j} \Psi _N \big \rangle \right| \nonumber \\&\quad \leqq 4 N^{-1/2} \left| \big \langle \nabla _j \Psi _N , a \big ( k B_{K,x_j} \big ) \Psi _N \big \rangle \right| + N^{-1} \left\| \Phi \big ( k B_{K,x_j} \big ) \Psi _N \right\| ^2 \nonumber \\&\quad \leqq 4 N^{-1/2} \left\| \nabla _j \Psi _N \right\| \left\| \left| \cdot \right| B_{K,x} \right\| \left\| {\mathcal {N}}^{1/2} \Psi _N \right\| + 4 N^{-1} \left\| \left| \cdot \right| B_{K,x} \right\| ^2 \left\| \left( {\mathcal {N}} + 1 \right) ^{1/2} \Psi _N \right\| ^2 \nonumber \\&\quad \leqq \sqrt{\frac{16 \pi }{K}} \big \langle \Psi _N , \left( - \Delta _j + N^{-1} {\mathcal {N}} \right) \Psi _N \big \rangle + \frac{16 \pi }{K} \big \langle \Psi _N , \frac{{\mathcal {N}} + 1}{N}\Psi _N \big \rangle . \end{aligned}$$

$$\square $$

The previous lemma readily implies the validity of the bounds (3.19) and (3.20). With

A.7 $$\begin{aligned} H_N^\mathrm{F} = H_N^0 + N^{-1/2}\sum _{j=1}^N \Phi (G_{\infty ,x_j}), \end{aligned}$$

we can use (A.1) with $\varepsilon = 1/2$ in order to infer (3.19). Using in addition (3.17) and (A.2), one similarly obtains (3.20).$\square$

**Comment on the proof of Lemma** 3.1.

As already explained, Lemma 3.1 was stated and proved in [16] for the case $N=1$. Since the statement $N\ge 2$ can be proven by almost literal adaption of the argument from [16] (with obvious minor modifications), we omit all details except for the proof of the following lemma. The bound given in the lemma is one of the main ingredients in the proof, and in particular its *N*-dependence is crucial since it guarantees that we can choose ${{\widetilde{K}}}$ in Lemma 3.1 independently of *N*.

### Lemma A.2

For any $\varepsilon >0$ there are $K_{\varepsilon } >0$ and $C_{\varepsilon } >0$ such that for all $N \in {\mathbb {N}}$, $K \geqq K_{\varepsilon }$ and any $\Psi _N \in {\mathcal {D}} ( H_N^0 )$,

A.8 $$\begin{aligned} \left\| \left( H_{N,K}^\mathrm{G} - H_N^0 \right) \Psi _N \right\| \leqq \varepsilon \left\| H_N^0 \Psi _N \right\| + C_\varepsilon K N \left\| \Psi _N \right\| . \end{aligned}$$

### Proof

We estimate each term in

A.9 $$\begin{aligned} H_{N,K}^\mathrm{G} - H_N^0&= \sum _{j=1}^N \left( N^{-1/2} \Phi (G_{K,x_j}) + A_{K,x_j} \right) + \sum _{j,l=1}^N V_{K}(x_j-x_l) \end{aligned}$$

separately. Using (3.4), $\left\| G_{K,x} \right\| \leqq C \sqrt{K}$ and $\left\| {\mathcal {N}}^{1/2} \Psi _N \right\| ^2 \leqq \left\| H_N^0 \Psi _N \right\| \left\| \Psi _N \right\|$, we have

A.10 $$\begin{aligned} N^{-1/2} \sum _{j=1}^N \left\| \Phi \left( G_{K,x_j} \right) \Psi _N \right\|&\leqq C \sqrt{K N} \left( \left\| {\mathcal {N}}^{1/2} \Psi _N \right\| + \left\| \Psi _N \right\| \right) \nonumber \\&\leqq \delta \left\| H_N^0 \Psi _N \right\| + C K N \left( 1 + \delta ^{-1} \right) \left\| \Psi _N \right\| \end{aligned}$$

for any $\delta >0$. The norm of $\sum _{j,l =1}^N V_{K}(x_j-x_l) \Psi _N$ can be bounded using (3.17). From (3.13) we see that the remaining terms to estimate are

A.11 $$\begin{aligned} N^{-1} \sum _{j=1}^N \left\| \Phi \left( k B_{K, x_j} \right) ^2 \Psi _N \right\|&\leqq C K^{-1} \left\| \left( {\mathcal {N}} + 1 \right) \Psi _N \right\| \nonumber \\&\leqq C K^{-1} \left( \left\| H_N^0 \Psi _N \right\| + \left\| \Psi _N \right\| \right) , \end{aligned}$$

where we have used (3.4) and (3.7). Similarly, we have

A.12 $$\begin{aligned} 2 N^{-1/2} \sum _{j=1}^N \left\| a^* \left( k B_{K, x_j} \right) \nabla _j \Psi _N \right\|&\leqq C \sqrt{N K^{-1}} \left\| \sqrt{{\mathcal {N}} + 1} \nabla _j \Psi _N \right\| \nonumber \\&\leqq C K^{-1/2} \left( \left\| H_N^0 \Psi _N \right\| + \left\| \Psi _N \right\| \right) \end{aligned}$$

and

A.13 $$\begin{aligned} 2 N^{-1/2} \sum _{j=1}^N \left\| \nabla _j a ( k B_{K, x_j} ) \Psi _N \right\|&\leqq 2 N^{-1/2} \sum _{j=1}^N \left( \left\| a ( k B_{K, x_j} ) \nabla _j \Psi _N \right\| + \left\| a ( \left| k \right| ^2 B_{K, x_j} ) \Psi _N \right\| \right) \nonumber \\&\leqq C K^{-1/2} \left\| H_N^0 \Psi _N \right\| + 2 N^{1/2} \left\| a ( \left| k \right| ^2 B_{K,x_1} ) \Psi _N \right\| . \end{aligned}$$

In order to estimate the second summand, we use

A.14 $$\begin{aligned} a^* ( \left| k \right| ^2 B_{K ,x_1} ) a ( \left| k \right| ^2 B_{K,x_1} ) \leqq {{\widetilde{C}}}_K (1-\Delta _1) {\mathcal {N}} , \end{aligned}$$

where

A.15 $$\begin{aligned} {\widetilde{C}}_K = \sup _{h \in {\mathbb {R}}^3} \int _{\left| k \right| \geqq K} d^3k \, \frac{ 1 }{ ( 1 + \left| k \right| ^2 ) \left( 1 + ( h - k \right) ^2 ) } . \end{aligned}$$

The bound (A.14) is analogous to (6.8) and can be proven in the same way as [10, Lemma 10] (see also [15, Lemma B.5]). If one estimates the integral in (A.15) using the Cauchy–Schwarz inequality one sees that ${{\widetilde{C}}}_K\rightarrow 0$ for $K\rightarrow \infty$. We thus have

A.16 $$\begin{aligned} 2 N^{1/2} \left\| a ( \left| k \right| ^2 B_{K,x_j} ) \Psi _N \right\|&\leqq 2 {\widetilde{C}}_K N^{1/2} \left\| \left( 1 - \Delta _j \right) ^{1/2} {\mathcal {N}}^{1/2} \Psi _N \right\| \nonumber \\&= 2 {\widetilde{C}}_K \big \langle \Psi _N , {\mathcal {N}} \sum \nolimits _{j=1}^N \left( 1 - \Delta _j \right) \Psi _N \big \rangle ^{1/2} \nonumber \\&\leqq {\widetilde{C}}_K \left( \left\| H_N^0 \Psi _N \right\| + N \left\| \Psi _N \right\| \right) , \end{aligned}$$

and hence,

A.17 $$\begin{aligned} 2 N^{-1/2} \sum _{j=1}^N \left\| \nabla _j a \left( k B_{K , x_j} \right) \Psi _N \right\|&\leqq \left( {\widetilde{C}}_K + C K^{-1/2} \right) \left( \left\| H_N^0 \Psi _N \right\| + N \left\| \Psi _N \right\| \right) . \end{aligned}$$

Choosing *K* large enough and $\delta$ sufficiently small completes the proof of the lemma. $\square$

## Time Derivative of $\beta ^b_K(t)$

Because of the unboundedness of the annihilation operator, it is not directly obvious that one can use the product rule of differentiation to obtain (6.15a) and (6.15b). Its rigorous justification relies on the estimate (for $\chi _N \in {\mathcal {D}}(H_N^0)$)

B.1 $$\begin{aligned} \left\| {\mathcal {N}} \chi _N \right\|&\leqq \left\| H_N^0 \chi _N \right\| \leqq 2 \left\| H_{N,K}^\mathrm{{G}} \chi _N \right\| + C K N \left\| \chi _N \right\| , \end{aligned}$$

which follows from Lemma A.2. Since $U_K \Psi _{N,t} = e^{-i H_{N,K}^\mathrm{{G}} t} U_K \Psi _{N,0}$, this together with the strong continuity of $e^{-i H_{N,K}^\mathrm{{G}} t}$ implies

B.2 $$\begin{aligned} \lim _{h \rightarrow 0} \left\| \left( {\mathcal {N}} + 1 \right) U_K \left( \Psi _{N,t+h} - \Psi _{N,t} \right) \right\| = 0 \quad \text {for all} \; U_K \Psi _{N,0} \in {\mathcal {D}} \left( H_N^0 \right) . \end{aligned}$$

Note that

B.3a $$\begin{aligned} \beta _K^b(\Psi _{N,t+h}, \varphi _{t+h}) - \beta _K^b(\Psi _{N,t}, \varphi _t)&= \beta _K^b(\Psi _{N,t+h}, \varphi _t) - \beta _K^b(\Psi _{N,t}, \varphi _t) \end{aligned}$$

B.3b $$\begin{aligned}&\quad + \beta _K^b(\Psi _{N,t+h}, \varphi _{t+h}) - \beta _K^b(\Psi _{N,t+h}, \varphi _{t}) \end{aligned}$$

and that the first line is given by

B.4 $$\begin{aligned} (\mathrm{B.3 a})&= 2 N^{-1} \mathrm {Re}\big \langle W ( \sqrt{N} \varphi _t ) {\mathcal {N}} W^*(\sqrt{N} \varphi _t) U_K \Psi _{N,t} , U_K \left( \Psi _{N,t+h} - \Psi _{N,t} \right) \big \rangle \nonumber \\&\quad + N^{-1} \big \langle W(\sqrt{N} \varphi _t) {\mathcal {N}} W^*(\sqrt{N} \varphi _t ) U_K \left( \Psi _{N,t+h} - \Psi _{N,t} \right) , U_K \left( \Psi _{N,t+h} - \Psi _{N,t} \right) \big \rangle . \end{aligned}$$

In the second line we use that

B.5 $$\begin{aligned}&\left\| {\mathcal {N}} W^*(\sqrt{N} \varphi _t) U_K \left( \Psi _{N,t+h} - \Psi _{N,t} \right) \right\| \nonumber \\&\quad \leqq C \left( 1 + N \left\| \varphi _t \right\| ^2 \right) \left\| \left( {\mathcal {N}} + 1 \right) U_K \left( \Psi _{N,t+h} - \Psi _{N,t} \right) \right\| \rightarrow 0 \end{aligned}$$

as $h \rightarrow 0$ because of (B.2) and obtain

B.6 $$\begin{aligned}&\lim _{h \rightarrow 0} h^{-1} \left( \beta _K^b(\Psi _{N,t+h}, \varphi _t) - \beta _K^b(\Psi _{N,t}, \varphi _t) \right) \nonumber \\&\quad = -2 N^{-1} \mathrm {Re}\big \langle W(\sqrt{N} \varphi _t) {\mathcal {N}} W^*(\sqrt{N} \varphi _t) U_K \Psi _{N,t} , i H_{N,K}^\mathrm{{G}} U_K \Psi _{N,t} \big \rangle \end{aligned}$$

with Stone’s theorem. Next, we consider

B.7a $$\begin{aligned} (\mathrm{B.3 b})&= - 2 \mathrm {Re}\int d^3k \, \big \langle \left( \varphi _{t+h}(k) - \varphi _t(k) \right) U_K \Psi _{N,t+h} , \left( N^{-1/2} a_k - \varphi _t(k) \right) U_K \Psi _{N,t+h} \big \rangle \end{aligned}$$

B.7b $$\begin{aligned}&\qquad + 2 \mathrm {Re}\int d^3k \, \big \langle \left( \varphi _{t+h}(k) - \varphi _t(k) \right) U_K \Psi _{N,t} , \left( N^{-1/2} a_k - \varphi _t(k) \right) U_K \Psi _{N,t} \big \rangle \end{aligned}$$

B.7c $$\begin{aligned}&\qquad + \left\| \varphi _{t+h} - \varphi _t \right\| ^2 \end{aligned}$$

B.7d $$\begin{aligned}&\qquad - 2 \mathrm {Re}\int d^3k \, \big \langle \left( \varphi _{t+h}(k) - \varphi _t(k) \right) U_K \Psi _{N,t} , \left( N^{-1/2} a_k - \varphi _t(k) \right) U_K \Psi _{N,t} \big \rangle . \end{aligned}$$

Using the Cauchy–Schwarz inequality we estimate the first two terms by

B.8 $$\begin{aligned}&\left| (\mathrm{B.7a}) + (\mathrm{B.7b}) \right| \nonumber \\&\quad \leqq 2 \int d^3k \, \left| \varphi _{t+h}(k) - \varphi _t(k) \right| \bigg ( \left| \big \langle U_K \Psi _{N,t+h} , \left( N^{-1/2} - \varphi _t(k) \right) U_K \left( \Psi _{N,t+h} - \Psi _{N,t} \right) \big \rangle \right| \nonumber \\&\qquad + \left| \big \langle U_K \left( \Psi _{N,t+h} - \Psi _{N,t} \right) , \left( N^{-1/2} a_k - \varphi _t(K) \right) U_K \Psi _{N,t} \big \rangle \right| \bigg ) \nonumber \\&\quad \leqq 2 N^{-1/2} \left\| \left( \varphi _{t+h} - \varphi _t \right) \right\| \bigg ( \left\| {\mathcal {N}}^{1/2} W^*(\sqrt{N} \varphi _t) U_K \left( \Psi _{N,t+h} - \Psi _{N,t} \right) \right\| \nonumber \\&\qquad + \left\| U_K \left( \Psi _{N,t+h} - \Psi _{N,t} \right) \right\| \left\| {\mathcal {N}}^{1/2} W^*(\sqrt{N} \varphi _t) U_K \Psi _{N,t} \right\| \bigg ). \end{aligned}$$

Stone’s theorem and (B.5) then lead to

B.9 $$\begin{aligned}&\lim _{h \rightarrow 0} h^{-1} \left( \beta ^b(\Psi _{N,t+h}, \varphi _{t+h}) - \beta _K^b(\Psi _{N,t+h}, \varphi _t) \right) \nonumber \\&\quad = - 2 \mathrm {Re}\int d^3k \, \big \langle \left( \partial _t \varphi _t(k) \right) U_K \Psi _{N,t} , \left( N^{-1/2} a_k - \varphi _t(k) \right) U_K \Psi _{N,t} \big \rangle . \end{aligned}$$

In combination, this shows (6.15a) and (6.15b).

## Footnotes

1 It should be noted that results about the polaron in the strong coupling limit are usually formulated in strong coupling units and that times of order $\alpha ^2$ in the stated references correspond to times of order one in the units of the present paper.

2 The Gross transform adds correlations between the bosons and phonon modes with momentum $\vert k \vert \ge K$. This leads to a better ultraviolet behavior of the radiation field.

3 From now on we omit the product with the identity and write $q_i^\psi$ and $p_i^\psi$ instead of $q_i^\psi \otimes \mathbb {1}_{{\mathcal {F}}_s}$ and $p_i^\psi \otimes \mathbb {1}_{{\mathcal {F}}_s}$.

4 We note that the quantity $\Vert \nabla _1 q_1^{\psi _t} U_{K} \Psi _{N,t} \Vert ^2$ can be related to the Sobolev trace norm difference between the one-particle reduced density matrix and the condensate wave function (see [27, Proof of Theorem 2.8] and [24, Lemma 7.1]).
